# Comparing the Effectiveness of Bevacizumab to Ranibizumab in Patients with Exudative Age-Related Macular Degeneration. The BRAMD Study

**DOI:** 10.1371/journal.pone.0153052

**Published:** 2016-05-20

**Authors:** A. M. E. Schauwvlieghe, G. Dijkman, J. M. Hooymans, F. D. Verbraak, C. B. Hoyng, M. G. W. Dijkgraaf, T. Peto, J. R. Vingerling, R. O. Schlingemann

**Affiliations:** 1 Department of Ophthalmology, Academic Medical Center, University of Amsterdam, Amsterdam, The Netherlands; 2 Ocular Angiogenesis Group, Departments of Ophthalmology and Cell Biology and Histology, Academic Medical Center, University of Amsterdam, Amsterdam, The Netherlands; 3 Department of Ophthalmology, Leiden University Medical Centre, Leiden, the Netherlands; 4 Department of Ophthalmology, University Medical Center Groningen, Groningen, the Netherlands; 5 Department of Ophthalmology, Radboud University Nijmegen Medical Centre, Nijmegen, The Netherlands; 6 Clinical Research Unit, Academic Medical Center, Amsterdam, The Netherlands; 7 National Institute for Health Research Biomedical Research Centre at Moorfields Eye Hospital and University College London Institute of Ophthalmology, Reading Centre, Moorfields Eye Hospital, London, the United Kingdom; 8 Department of Ophthalmology, Erasmus Medical Center, Rotterdam, The Netherlands; 9 Department of Epidemiology, Erasmus Medical Center, Rotterdam, The Netherlands; 10 Department of Biomedical Engineering and Physics, Academic Medical Center, University of Amsterdam, Amsterdam, The Netherlands; 11 Netherlands Institute for Neurosciences, Amsterdam, The Netherlands; Medical University Graz, AUSTRIA

## Abstract

**Purpose:**

To compare the effectiveness of bevacizumab and ranibizumab in the treatment of exudative age-related macular degeneration (AMD).

**Design:**

Multicentre, randomized, controlled, double-masked clinical trial in 327 patients. The non-inferiority margin was 4 letters.

**Patients:**

Patients ≥ 60 years of age with primary or recurrent sub- or juxtafoveal choroidal neovascularization (CNV) secondary to AMD with a total area of CNV < 12 disc areas and a best corrected visual acuity (BCVA) score between 20 and 78 letters on an EDTRS like chart in the study eye.

**Methods:**

Monthly intravitreal injections with 1.25 mg bevacizumab or 0.5 mg ranibizumab were given during one year. Intention to treat with last observation carried forward analysis was performed.

**Main Outcome Measures:**

Primary outcome was the change in BCVA in the study eye from baseline to 12 months.

**Results:**

The mean gain in BCVA was 5.1 (±14.1) letters in the bevacizumab group (n = 161) and 6.4 (±12.2) letters in the ranibizumab group (n = 166) (p = 0.37). The lower limit of the 95% confidence interval of the difference in BCVA gain was 3.72. The response to bevacizumab was more varied; 24% of patients showed a gain of ≥15 letters, 11% a loss of ≥15 letters and 65% a gain or loss < 15 letters compared to 19%, 5% and 76% respectively for ranibizumab (p = 0.038). No significant differences in absolute CRT and CRT change (p = 0.13) or in the presence of subretinal or intraretinal fluid (p = 0.14 and 0.10, respectively) were observed. However, the presence of any fluid on SD-OCT (subretinal and/or intraretinal) differed significantly (p = 0.020), with definite fluid on SD-OCT in 45% of the patients for bevacizumab versus 31% for ranibizumab. The occurrence of serious adverse events and adverse events was similar, with 34 SAEs and 256 AEs in the bevacizumab group and 37 SAEs and 299 AEs in the ranibizumab group (p = 0.87 and p = 0.48, respectively).

**Conclusions:**

Bevacizumab was not inferior to ranibizumab. The response to bevacizumab was more varied with higher percentages of both gainers and losers and more frequently observed retinal fluid on SD-OCT at 12 months when compared to the ranibizumab group.

**Trial Registration:**

Trialregister.nl NTR1704

## Introduction

Exudative age-related macular degeneration (exudative AMD) is the main cause of untreatable blindness in western countries and a major burden for the elderly population [1,2]. Standard treatment of exudative AMD is with intravitreal injections of vascular endothelial growth factor (VEGF) antagonists. These treatments maintain vision in up to 90 percent of patients but do not cure AMD. [3]

The most commonly used VEGF-antagonists are bevacizumab (Avastin ^®,^ Genentech/Hoffmann-La Roche), ranibizumab (Lucentis^®^, Genentech/Novartis, Inc.) and aflibercept (Eylea^®,^, Bayer). The active part of the molecule is similar in bevacizumab and ranibizumab. However, bevacizumab is the whole anti-VEGF antibody (150 kD), while ranibizumab is an antibody fragment. Bevacizumab has a longer half-life in the systemic circulation than ranibizumab while ranibizumab is believed to penetrate the retina better and has higher affinity to VEGF-A than bevacizumab. These differences could have an impact on safety and efficacy of these drugs [4].

Since 2008, several comparative clinical trials were initiated to compare bevacizumab and ranibizumab in exudative AMD. Recently three of these reported bevacizumab not to be inferior to ranibizumab in the treatment of AMD [4–6] while one trial concluded bevacizumab to be neither non-inferior nor inferior to ranibizumab at the two-year endpoint.[7]. The CATT study was a head-to-head comparison of bevacizumab and ranibizumab in 1,208 AMD patients using both monthly and ‘as needed’ (Pro Re Nata, PRN) treatment regimens (4 groups). With the non-inferiority limit set at 5 letters, they found distance visual acuity (VA) after 1 year to be equivalent for the two drugs within each of these treatment regimens. [6] The IVAN study in 610 patients had a similar study design (4 groups), although the criteria and regimen of retreatment in the PRN groups were different from CATT, and the non-inferiority limit was 3.5 letters. When patients treated with bevacizumab (monthly and PRN) were compared with one-sided testing (p<0.05), to all patients treated with ranibizumab, bevacizumab was neither non-inferior nor inferior to ranibizumab. Other efficacy and safety outcomes were similar between groups, such as retinal thickness using time-domain Optical Coherence Tomography (OCT). [7] The MANTA research group and the GEFAL research group included 317 and 501 patients respectively. They compared both drugs in PRN treatment regimens (2 groups). [4,5] In the MANTA trial, again bevacizumab was non-inferior to ranibizumab for VA at all time points over 1 year. No significant differences were found in decrease of retinal thickness or in number of adverse events. [4] In the GEFAL study, the difference in mean change in best corrected visual acuity favored bevacizumab with 1.68 letters (p <0.0001, 95% confidence interval, -1.16 to +4.93). [5]

The BRAMD study is the second study comparing the efficacy and costs of a regimen of monthly intravitreal injections of bevacizumab and ranibizumab. Patients with new or recurrent exudative AMD were treated for one year in a multicenter study in The Netherlands.

## Methods

### Study Design, Participants and Setting

The BRAMD trial is a triple masked, randomized, clinical non-inferiority trial. All patients received monthly injections for 12 months. Between January 2009 and December 2011, 327 patients were recruited at 5 academic medical centres in the Netherlands. There was approval by the institutional ethical review board at each centre. The study adhered to the tenets of the Declaration of Helsinki. Eligible patients were 60 years of age or older with primary or recurrent sub- or juxtafoveal choroidal neovascularization (CNV) secondary to AMD with a total area of CNV of < 12 disc areas and a best corrected visual acuity (BCVA) score between 20 and 78 letters on an Early Treatment Diabetic Retinopathy Study- (ETDRS) like chart in the study eye. In- and exclusion criteria are listed in Table 1.

**Table 1:** In- and exclusion criteria.

Inclusion criteria
Patients 60 years of age or higher.
Patients with primary or recurrent sub-, juxta- or extrafoveal CNV secondary to AMD, including those with RAP, that may benefit from anti-VEGF treatment in the opinion of the investigator.
Patients with primary or recurrent sub-, juxta- or extrafoveal CNV secondary to AMD, including those with RAP, that may benefit from anti-VEGF treatment in the opinion of the investigator.
The total area of CNV (including both classic and occult components) encompassed within the lesion must be more or equal to 30% of the total lesion area.
The total lesion area should be < 12 disc areas.
A best corrected visual acuity (BCVA) score between 78 and 20 letters (approximately 0,63–0,05 Snellen equivalent) in the study eye.
Exclusion criteria
Ocular treatment with anti-angiogenic drugs in the last 2 months or Triamcinolone in the last 6 months.
Laser photocoagulation (juxtafoveal or extrafoveal) in the study eye within one month preceding Baseline.
Patients with angioid streaks or precursors of CNV in either eye due to other causes, such as ocular histoplasmosis, trauma, or pathologic myopia.
Spherical equivalent of refractive error in the study eye demonstrating more than– 8 dioptres of myopia.
Cataract extraction within three months preceding Baseline
IOP >25 mm Hg
Active intraocular inflammation in the study eye.
Vitreous haemorrhage obscuring view of the posterior pole in the study eye.
Presence of a retinal pigment epithelial tear involving the macula in the study eye.
Subretinal haemorrhage in the study eye if the size of the haemorrhage is > 70% of the lesion
Subfoveal fibrosis or atrophy in the study eye.
History of hypersensitivity or allergy to fluorescein.
Inability to obtain fundus photographs, fluorescein angiograms or OCT’s of sufficient quality to be analyzed and graded by the Central Reading Centre.
Systemic disease with a life expectancy shorter than the duration of the study.
Inability to adhere to the protocol with regard to injection and follow-up visits.
Legally incompetent adult
Refusal to give written informed consent

Decisions about eligibility were made based on Fluorescein Angiograms (FA), fundus photography and Spectral Domain Optical tomography (SD-OCT). Diagnosis and presence of active CNV due to AMD was confirmed by independent graders at the UK Network of Ophthalmic Reading Centers. The study is registered at the Dutch trial register (Nederlands trial register) (NTR1704).

### Interventions

After informed written consent, participants were allocated to one of two study arms: monthly injections (window, 30 ± 7 days) with 1.25 mg of bevacizumab or with 0.5 mg ranibizumab. The commercially available formulations of bevacizumab and ranibizumab were used and both were prepared for injection by aspiration in a Kendall monoject syringe in an aseptic manufacturing facility to ensure masking for everybody taking part in the study, apart from the pharmacists. Syringes were only labelled with the patient identification number. Prepared syringes were kept at 4°Celsius and injections were given not later than 24 hours after preparation. Participants attended monthly for a protocolized BCVA measurement, SD-OCT (3D and cross scans) and intravitreal injection with the allocated drug. Besides the identical syringes masking was also ensured by the fact that the ophthalmologists who performed the injections did not take part in interpretation of any data or patient assessment. The patient was labelled as a poor-responder and treatment was changed to the other drug, if at any visit after the third injection there was a drop in BCVA of more than 10 letters compared to baseline and there was clear evidence of active CNV or leakage by qualitative SD-OCT and/or FA assessment or at least two of the following signs of leakage on OCT; central retinal thickening >300 micron (CRT), intraretinal cysts or subretinal fluid any time after the third injection. The choice for CRT > 300 micron was based on the assumption that this would be more than two standard deviations above the mean CRT of a healthy retina in all three the devices used (see also below). FA and a standardized full ophthalmic examination were done at baseline, 4 months and exit visit.

### Outcome Measures

The primary outcome was the change in BCVA in the study eye from baseline to month 12 assessed with ETDRS- like visual acuity charts at an initial distance of four meter. Secondary outcome measures were: the proportion of patients with a loss of BCVA less than 15 letters from baseline at 12 months (responders); the proportion of patients with a loss or a gain of BCVA less than 15 letters from baseline at 12 months (stabilizers); the proportion of patients with 15 letters loss or more of BCVA from baseline at 12 months (losers); the proportion of patients with 15 letters gain or more of BCVA from baseline at 12 months (gainers); the absolute and percentage change in CRT, as measured by SD-OCT at 4 and 12 months as determined by the Study Reading Centre at the AMC; the proportion of dropouts before the final 12 months assessments; the proportion of switchers after the third injection; the occurrence of (serious) adverse events during the 12 months of the study and the costs of the two treatments. The latter will be discussed in the companion paper.

For SD-OCT examinations, either Cirrus OCT, Spectralis OCT or Topcon OCT could be used. The type of SD-OCT device used differed between sites, but individual patients were always scanned with the same device during the study. Although the different devices have different definitions for the inner- and outer border of the retina, absolute differences were assumed not to differ. Additionally, all volume scans were reviewed by two investigators to check for proper positioning on the macula as well as consistency of detection of the inner and outer border of the retina. Where necessary, borders were adjusted manually. Data on injections with bevacizumab or ranibizumab and data on imaging and other therapeutic procedures were gathered with case report forms.

Trial coordinators questioned patients at each visit regarding adverse events and the events were coded by one researcher at the coordinating site using Medical Dictionary for Regulatory Activities (MeDdra 14.1) system. All serious adverse events were reviewed by the principal investigator.

### Sample size calculation

Differences in the BCVA change scores from baseline were tested statistically for non-inferiority. Starting from a common standard deviation of the change in BCVA score of 14 letters in both groups, and assuming an improvement from baseline of 9 letters in both the ranibizumab and bevacizumab groups, a sample size of 306 patients (153 in each group) has an 80% power of demonstrating non-inferiority by excluding a difference of 4 letters or more, using a one-sided t-test and a significance level of 0.05.

### Randomization and Masking

The randomisation list was created in a 1:1 ratio by the TENALEA Clinical Trial Data Management System. The allocation scheme was stratified by centre, by BCVA in the study eye (≤52 versus ≥53) and by BCVA in the fellow eye (≤52 versus ≥53) using the (non-deterministic) minimization method as described by Pocock and Simon [8] in each substratum. The randomization list was imported into the data management system Oracle Clinical. Upon randomization of a patient, an automatized email notification containing the allocation result was sent to the site's pharmacy keeping the investigator and trial personnel blinded from treatment allocation. In case a medication switch was requested in Oracle Clinical, an automatized e-mail notification containing the updated treatment allocation was sent to the site's pharmacy.

### Statistical Analysis

Non-inferiority is assumed if the difference between both groups is 4 letters or less using a one-sided t-test with a significance level of 0.05. Based on the literature available before January 2009, we set the non-inferiority margin at 4 letters. We performed intention-to-treat (ITT) analysis. When patients did not complete the study, their last available BCVA-score was used as the BCVA-score at visit 14 (last-observation-carried-forward). Further, to minimize the risk of false claiming non-inferiority we used the BCVA at the moment of switch for patients who were switched to the other treatment. The mean BCVA-change per treatment group was calculated. Covariance analysis of the BCVA-change was used with treatment as fixed factor and baseline BCVA-score as covariate.

To evaluate the influence of carrying forward the last available BCVA-score to visit 14 in patients without BCVA-score at visit 14, we used a linear mixed-effects regression analysis to analyze the repeatedly measured BCVA-change from baseline. In this model we used treatment, visit-number and their interaction as fixed-factors and the baseline BCVA-score as covariate. We used cubic-splines to flexibly model the change of the BCVA over time. No assumptions concerning the covariance matrix between the repeated BCVA-scores were made. The 90% confidence interval of the difference between the estimated means of BCVA-change from baseline at visit 14 in the two treatment groups was calculated.

The absolute and percentage change from baseline in CRT, as measured by SD-OCT, at 4 and 12 months, were analyzed with covariance analysis. The significance level was 0.05 and last-observation-carried-forward was performed. Only measurements by the Study Reading Centre Amsterdam were used.

All proportions were compared between treatment groups using the Pearson chi-square statistic with a significance level of 0.05. These proportions were calculated after last-observation-carried-forward in patients without BCVA-score at visit 14.

The numbers of adverse events and serious adverse events during the 12 months of the study were compared between the treatment groups using the Mann-Whitney U test using a significance level of 0.05.

## Results

### Patients

**Fig 1:**
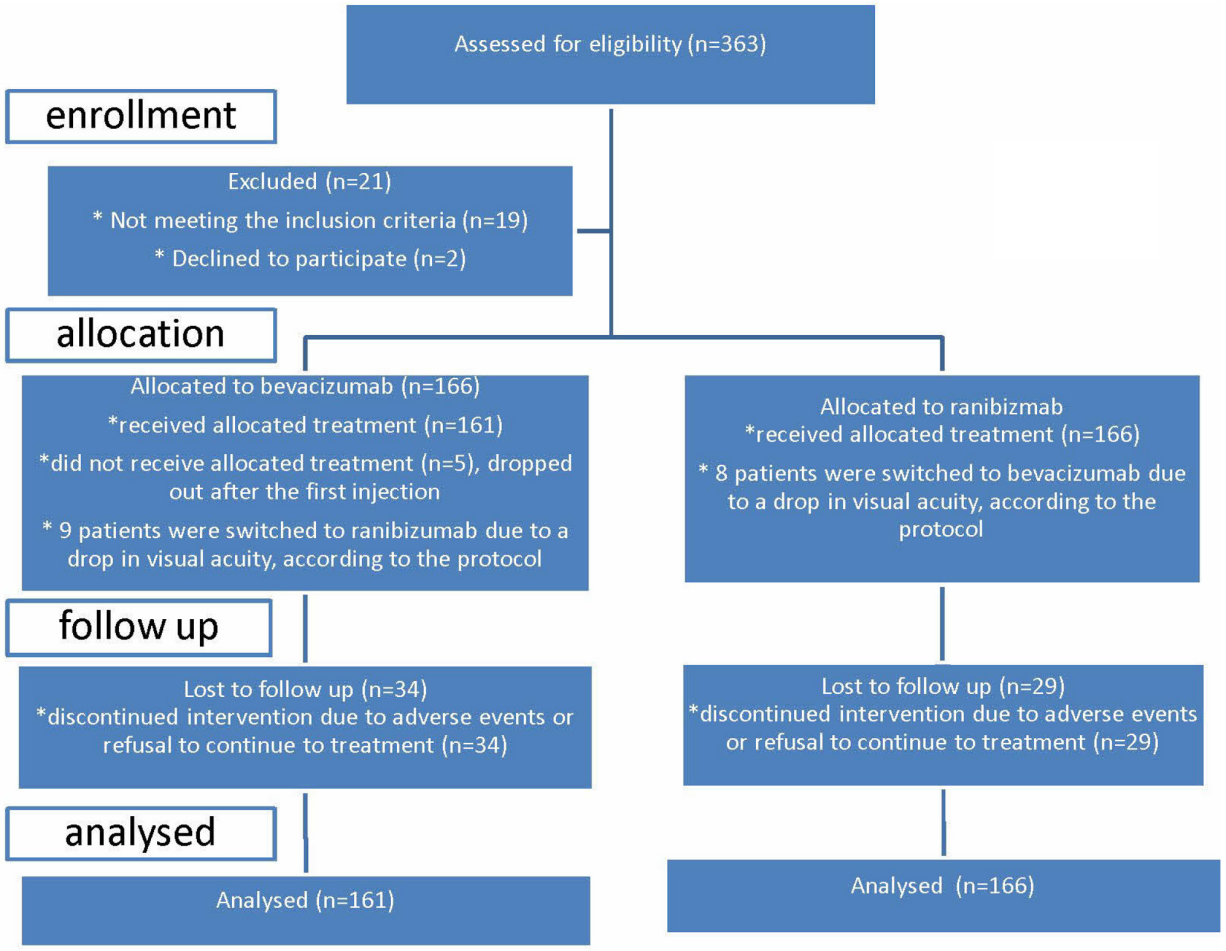
Consort flow chart.

Between January 2009 and December 2011 we randomized 332 patients. The consort flow chart can be seen in Fig 1. Five patients were excluded from the study before the second injection and were excluded from further analyses because there was no effect measurement. There were no substantial imbalances in the demographic or ocular characteristics of both treatment arms at baseline (Table 2).

**Table 2:** Baseline Characteristics.

Characteristic	Bevacizumab (n = 161)	Ranibizumab (n = 166)	all (n = 327)
Age (yr): mean (SD)	79 (7)	78 (7)	78 (7)
Gender–male: n (%)	72 (45%)	73 (44%)	145 (44%)
Caucasian: n(%)	158 (98%)	163 (98%)	321 (98%)
Non caucasian: n (%)	3 (2%)	3 (2%)	6 (2%)
Mean Best Corrected Visual Acuity: mean (SD)	60 (13)	60 (14)	60 (13)
BCVA ≤ 52 letters: n (%)	42 (26%)	43 (26%)	85 (26%)
BCVA ≥ 53 letters: n (%)	119 (74%)	123 (72%)	242 (74%)
Central retinal thickness (micron): mean (SD)	383 (119)	374 (113)	378 (115)
Tonometry	14.89 (2.97)	15.05 (3.10)	14.98 (3.03)
No Foveal center involvement: n (%),	75 (47%)	77 (46%)	152 (47%)
Active CNV: n (%)	161 (100%)	165 (99.9%)	326 (99.9%)
PED: n (%)	59 (43%)	61 (42%)	120 (42%)
Lesion area* (disc areas): mean (SD)	2.77 (2.16)	2.67 (2.12)	2.72 (2.13)
Lesion area* (disc areas): median (min-max)	2 (0–11)	2 (0–11)	2 (0–11)
Predominantly Classic CNV *: n (%)	44 (28%)	41 (26%)	85 (27%)
Minimally Classic CNV *: n (%)	18 (12%)	33 (21%)	51 (16%)
Occult CNV *: n (%)	93 (60%)	84 (53%)	177 (57%)
Subretinal fluid*: absent n (%)	19 (12%)	12 (7%)	31 (10%)
Subretinal fluid*: definite n (%)	132 (83%)	141 (86%)	273 (84%)
Subretinal fluid*: questionable n (%)	9 (6%)	10 (6%)	19 (6%)
Subretinal fluid*: Can’t grade n (%)	-	1 (1%)	1 (1%)
Intraretinal cysts*: absent n (%)	54 (34%)	51 (31%)	105 (32%)
Intraretinal cysts*: definite n (%)	88 (53%)	90 (55%)	175 (54%)
Intraretinal cysts*: questionable n (%)	21 (13%)	23 (14%)	44 (14%)
subretinal fluid or intraretinal cysts: absent n (%)	-	3 (2%)	3 (1%)
subretinal fluid or intraretinal cysts: definite: n (%)	154 (96%)	158 (96%)	312 (96%)
subretinal fluid or intraretinal cysts: questionable: n (%)	6 (4%)	3 (2%)	9 (3%)
History of myocardial infarction: n (%)	17 (11%)	18 (11%)	35 (11%)
History of stroke: n (%)	2 (1%)	5 (3%)	7 (2%)
History of transient ischemic attack: n (%)	13 (8%)	16 (10%)	29 (9%)
History of angina pectoris: n (%)	21 (13%)	22 (13%)	43 (13%)
History of dyspnea: n (%)	25 (15%)	24 (14%)	49 (15%)
History of Thrombosis: n (%)	6 (4%)	5 (3%)	11 (3%)
Systolic Blood pressure (mmHg): mean (SD)	150 (23)	155 (22)	152 (23)
Diastolic Blood pressure (mmHg): mean (SD)	80 (11)	82 (11)	81 (11)
EQ-5D state score: mean (SD)	6.2 (1.2)	6.4 (1.3)	6.3 (1.3)
Pseudophakia: n (%)	64 (40%)	68 (41%)	132 (40%)

* = as judged by the local investigator

National Institute for Health Research Biomedical Research Centre at Moorfields Eye Hospital and University College London Institute of Ophthalmology; Reading Centre, Moorfields Eye Hospital confirmed presence of active CNV due to AMD in all but one patient. There were a few other protocol violations: injection other than the masked study medication was administered during 7 visits, with a probability of 0.5 of having injected the wrong substance. Other protocol violations were missed visits due to AEs or SAEs (n = 22), visits where no injection was given for patient’s safety reasons (n = 5), and inability to obtain SD-OCT or FA.

### Visual Acuity

**Fig 2:**
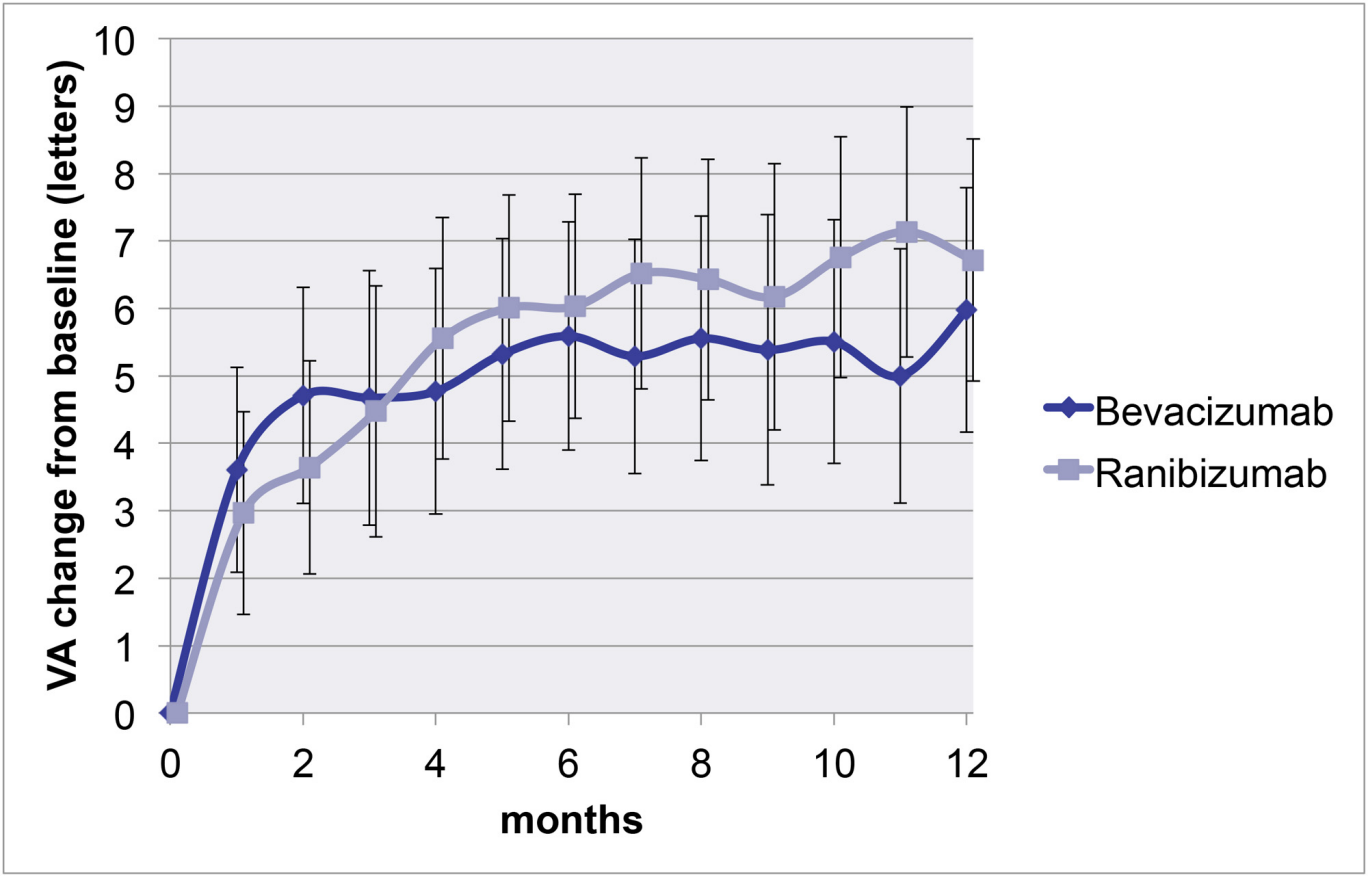
Mean change in BCVA from baseline. Mean change in BCVA per group. A mixed linear model was used for this graph. This type of analysis shows a mean gain of 5.9 letters in the bevacizumab group and a mean gain of 6.7 letters in the ranibizumab group (p = 0.56).

All patients receiving more than one injection were included in the BCVA analysis. At 1 year, mean gain in BCVA was 5.1 (SD = 14.1) letters in the bevacizumab group and 6.4 (SD = 12.2) letters in the ranibizumab group (p = 0.37). The lower limit of the 95% confidence interval of the difference in BCVA gain between the two groups was 3.72. Because this is smaller than 4 letters, we can conclude that bevacizumab is not inferior to ranibizumab (Fig 2).

### Secondary outcomes

The response to bevacizumab was significantly more varied than to ranibizumab as in the bevacizumab group 24% of patients had a gain of ≥15 letters, 11% a loss of ≥15 letters and 65% a gain or loss < 15 letters whereas in the ranibizumab group this was 19%, 5% and 76%, respectively (p = 0.038).

The proportions of patients with a BCVA ≥ 20/40 remained similar between groups throughout the whole study: 27% at baseline (43 of 161 patients) in the bevacizumab group versus 29% (48 of 166) in the ranibizumab group. At exit these proportions increased to 56% (90 out of 161) and 54% (89 out of 166), respectively (p = 0.68).

**Fig 3:**
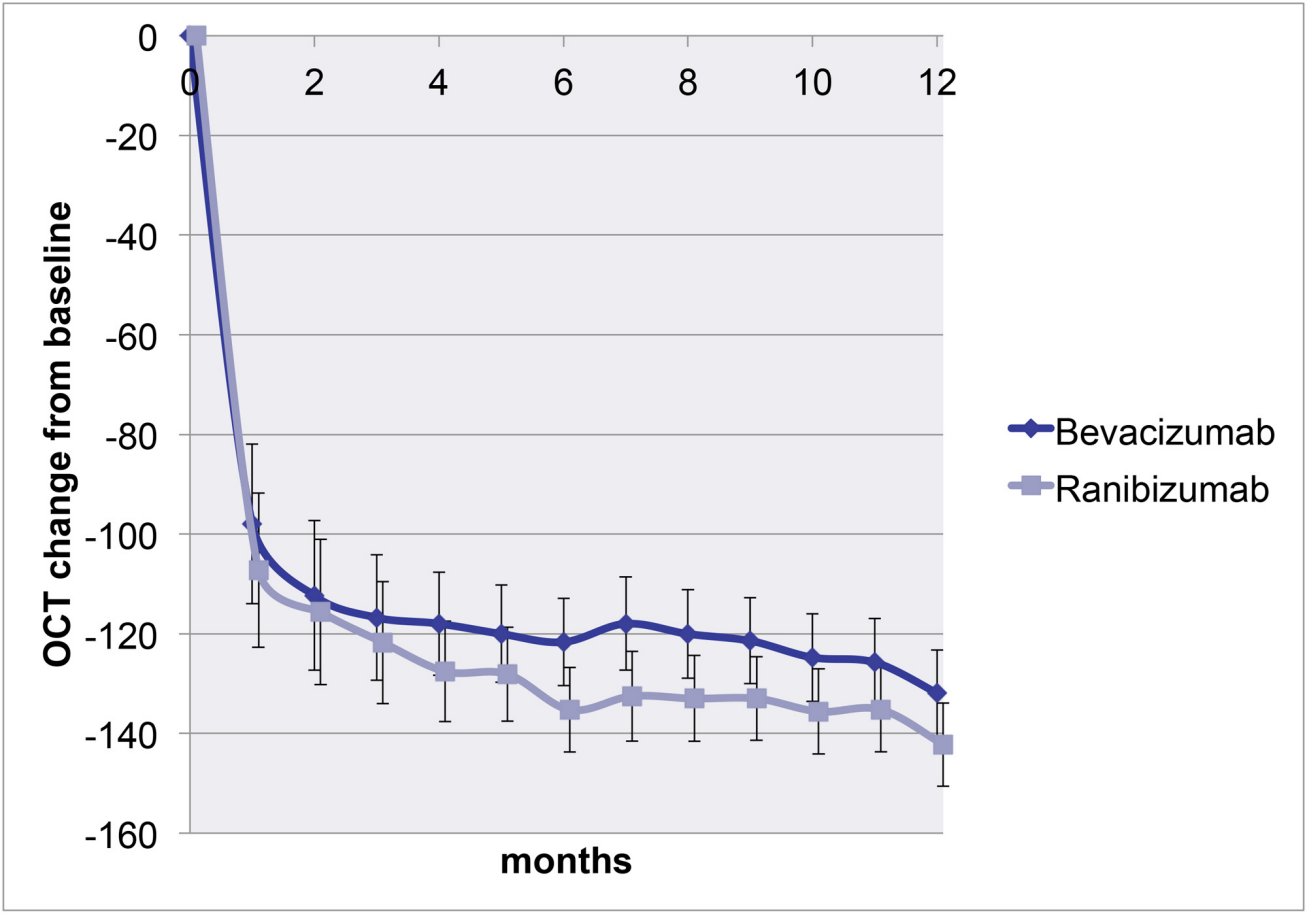
Mean change in central retinal thickness. Effect of bevacizumab (n = 161, blue) versus ranbizumab (n = 166, red) on the change of retinal thickness over time. Data are presented as mean ±95% CI. A mixed linear model was used for this graph.

On SD-OCT, no significant differences in CRT at 12 months were found, with a CRT for bevacizumab of 258 (SD = 78) micron and for ranibizumab of 246 (SD = 62) micron (p = 0.13). The change in CRT at exit also did not differ between both drugs, with a mean decrease of 131 (SD = 129) micron in the bevacizumab group and 138 (SD = 117) micron in the ranibizumab group, (p = 0.31). Fig 3. There were no significant differences in mean change in CRT when comparing the devices. Mean change in CRT was a decrease of 128 micron (SD = 115) for the Cirrus, 134 (SD = 115) for the Topcon and 124 micron (SD = 107) for the Spectralis OCT (p = 0.89).

There were no statistically significant differences at exit in the presence of subretinal or intraretinal fluid, as judged by local investigators, when evaluated separately (p = 0.14 and 0.10 respectively). However, for the parameter ´any fluid (subretinal and/or intraretinal) on SD-OCT´ there was a significant difference (p = 0.020), with definite fluid on SD-OCT in 45% of the patients at exit in the bevacizumab group versus 31% in the ranibizumab group, and questionable fluid in 11% in the bevacizumab group versus 10% in the ranibizumab.

As a rescue treatment, patients showing insufficient response to treatment were switched from the originally assigned treatment to the other based on strictly predefined criteria. Nine patients originally treated with bevacizumab were switched to ranibizumab. The opposite switch was done in eight patients. Further analysis of these patients showed that not all criteria for switch were met in 8 out of these 17 patients. Analysis excluding all switchers shows a mean change in BCVA of 6.64 (SD = 12.8) in the bevacizumab group and 7.11 (SD = 11.6) letters in the ranibizumab group (p = 0.77).

Thirty-four patients dropped out in the bevacizumab group (21%) compared to 29 (17%) in the ranibizumab group. Not considering the switchers as drop outs would reduce these numbers to 25 (16%) and 21 (13%) respectively (p = 0.53%).

Excluding all dropouts, hence performing a per protocol analysis, results to mean changes in BCVA of 7.28 (SD = 10.5, n = 127) and 6.47 (SD = 10.77, n = 137) (p = 0.50), resulting in a small advantage for bevacizumab.

Our study population differed from the other clinical trials since not all patients included in the study were treatment naive. Forty-three patients had received previous treatment for their exudative AMD. Nineteen were randomized in the bevacizumab group (12%) and 24 in the ranibizumab group (15%) (p = 0.51). Of these 43 patients 4 were switched; 3 from ranibizumab to bevacizumab and one from bevacizumab to ranibizumab. Hence 9% of patients previously treated were switchers compared to 5% of the treatment naïve patients (p = 0.26).

Patients with a history of treatment had a higher mean BCVA at baseline (61.67, SD = 13.18) compared to the treatment naïve patients (59.39, SD = 13.37) (p = 0.30). Mean gain in BCVA for previously treated patients was 1.21 (SD = 12.30) compared to 6.44 (SD = 13.15) for the treatment naïve patients(p = 0.015).

The mean change in BCVA in previously treated patients differed 1.44 (SE1.44) in favour of ranibizumab, but the limit of the confidence interval is -3.83, i.e. smaller than 4 letters (p = 0.32).

There is no significant interaction between previous treatment and either treatment arm (p = 0.22).

Treatment naïve patients had a mean change in BCVA of 6.06 (SD = 13.67) letters in the bevacizumab group and 6.82 letters (SD = 12.63) in the ranibizumab group (p = 0.63). In previously treated patients mean change in BCVA decreases 2.11 letters (SD = 15.38) and increases 3.83 letters(SD = 8.66) respectively (p = 0.13). (Table 3)

**Table 3:** Outcome Measures at 12 months.

	Bevacizumab (n = 161)	Ranibizumab (n = 166)	P—value
Best Corrected visual acuity, letters: mean (SD)	65.0 (19.0)	66.4 (15.8)	0.37
Mean gain in BCVA (SD)	5.09 (14.09)	6.40 (12.17)	0.37
Gainers vs losers vs staibilizers			0.038
Gainers (= % with gain ≥ 15 letters)	39 (24%)	32 (19%)	
Losers (= % with loss ≥ 15 letters)	18 (11%)	8 (5%)	
Stabilizers (= % with loss or gain < 15 letters)	104 (65%)	126 (76%)	
Responders (= % with loss < 15 letters)	143 (89%)	158 (95%)	0.041
Central retinal thickness (CRT)	258 (78)	246 (62)	0.13
Absolute change in CRT: mean (SD)	-131 (129)	-138 (117)	0.31
% change in CRT: mean (SD)	-30 (23)	-32 (19)	0.13
Tonometry: mean (SD)	14.01 (3.18)	15.05 (3.05)	0.001
Absolute change in tonometry: mean (SD)	-0.93 (2.97)	-0.06 (2.67)	0.001
Subretinal fluid* absent vs definite, vs questionable			0.14
Subretinal fluid*: absent n (%)	101 (63%)	121 (73%)	
Subretinal fluid*: definite n (%)	45 (28%)	35 (21%)	
Subretinal fluid*: questionable n (%)	15 (9%)	6%)	
intraretinal cysts* absent vs definite, vs questionable			0.10
Intraretinal cysts*: absent n (%)	104 (65%)	117 (71%)	
Intraretinal cysts*: definite n (%)	40 (25%)	26 (16%)	
Intraretinal cysts*: questionable n (%)	17 (11%)	23 (14%)	
subretinal fluid or intraretinal cysts absent vs definite, vs questionable			0.020
subretinal fluid or intraretinal cysts absent: n (%)	71 (44%)	98 (59%)	
subretinal fluid or intraretinal cysts: definite n (%)	72 (45%)	51 (31%)	
subretinal fluid or intraretinal cysts: questionable n (%)	18 (11%)	17 (10%)	
Active CNV*	58 (36%)	56 (34%)	0.73
Type of lesion* predominantly classic vs minimally classic vs occult			0.035
Predominantly Classic CNV*	38 (24%)	40 (24%)	
Minimally classic CNV*	17 (11%)	34 (21%)	
Occult CNV*	106 (66%)	92 (55%)	
Blood pressure Systolic: mean (SD)	148 (22)	151 (22)	0.71
Diastolic pressure Systolic: mean (SD)	79 (11)	81 (11)	0.48
Number of switchers	9 (6%)	8 (5%)	0.81

* As judged by local investigator

The number of serious adverse events was similar in both groups; 34 SAEs in the bevacizumab group compared to 37 in the ranibizumab group (p = 0.87). Occurrence of non-serious adverse events was also not statistically significant between groups; 256 AEs for the bevacizumab group versus 299 in the ranibizumab group (p = 0.48). Tables 4 and 5.

**Table 4:** Serious adverse events.

Occurrence of SAEs	34	37	0.87
Death due to SAE	1	1	
Life-threatening condition	1	2	
hospitalisation	30	32	
severe or permanent damage	1	0	
no relation to study medication	32	35	
improbable relation to study medication	1	1	
Occurrence of AEs	256	299	0.48

**Table 5:** Adverse events by organ system.

MedDRA system organ class	Bevacizumab (n = 49)	percentage	Ranibizumab (n = 52)	percentage
Cardiac disorder	4	2,5	6	3,6
Infection	4	2,5	4	2,4
Nervous system disorder	3	1,9	1	0,6
Injury or procedural complication	5	3,1	1	0,6
Benign or malignant neoplasm	2	1,2	3	1,8
Surgical or medical procedure	13	8,1	16	9,6
Gastrointestinal disorder	2	1,2	2	1,2
Any other system organ class	18	11,2	17	10,2

There were no statistically significant difference regarding serious adverse events. However, the study was underpowered to properly analyze safety.

The numbers of SAEs reported here seem higher than mentioned above this is because some SAEs are related to several groups.

## Discussion

Like the CATT trial [6], the BRAMD study demonstrates that the efficacy of monthly treatment of exudative age-related macular degeneration with intravitreal injections of bevacizumab is not inferior to monthly treatment with ranibizumab. Although the design and outcome of the BRAMD study were similar to those from the CATT and IVAN trials in general terms, they differed in detail [6,7]. The BRAMD study is the first fully masked trial comparing bevacizumab and ranibizumab in exudative AMD, as injecting physicians were masked to the medication administrated.

In addition, in contrast to the CATT and IVAN studies, the BRAMD study also included patients with recurrent CNV. This seems to contribute to the slightly lower gain in visual acuity observed in both treatment arms of the BRAMD study.

Consistent with the results of CATT, IVAN and MANTA studies, the mean treatment outcome with bevacizumab showed slightly less gain in BCVA, but of a magnitude that can be regarded as clinically insignificant [4,6,7]. However, in the BRAMD study, the response to bevacizumab was markedly more varied than the response to ranibizumab, as shown by the distribution of ´stabilizers´, ´gainers´ and ´losers´ in both groups. The proportions of gainers (≥ 15 letters gain) and losers (≥15 letters loss) were higher in the bevacizumab group at 24% and 11% versus 19% and 5% in the ranibizumab group, respectively. The larger spread in response can also be deduced from the larger standard deviation of change in visual acuity of this group (14 vs. 12). These results suggest that, although the overall mean outcome of bevacizumab and ranibizumab do not differ, the response to bevacizumab is more varied than to ranibizumab. This is clinically significant, as twice as many patients experienced substantial vision loss in the bevacizumab arm of the BRAMD trial as in the ranibizumab group. The percentage of patients with substantial vision loss in the ranibizumab group was consistent with previous results of the ANCHOR and MARINA trials, where 95% responded. In the bevacizumab group of the BRAMD study however, we identified only 89% responders. [9,10]

The IVAN study has not reported the distribution of gainers and losers, but in the CATT trial 31.3% gainers, 62.7% stabilizers and 6% losers in the bevacizumab monthly group versus 34.2%, 60.2% stabilizers and 5.6% in the ranibizumab monthly group were observed [6]. They had 62.7% stabilizers in the bevacizumab monthly group versus 60.2% in the ranibizumab monthly group. These results are not consistent with the BRAMD study outcomes, but the different outcomes between our study and CATT could be due to several differences in the study design. The CATT protocol of BCVA measurements entailed e-EDTRS charts where computer screens were used and the protocol was semi-automated whereas we used refraction with analog charts by technicians. CATT included patients with a baseline BCVA between 23 and 82 letters whereas in the BRAMD this was 20 and 78 letters. Minimum time between injections in CATT protocol was 21 days whereas in our protocol treatment window was from 23 to 37 days. In addition, we included patients with new or recurrent disease and 43 of our patients had been treated previously whereas in CATT this was an exclusion criterion. More importantly, the CATT protocol allowed the treating physician to refrain from further injections for patients thought to unlikely benefit from further injections, whereas in our protocol patients with poor outcome were switched to the other drug.

Like in the CATT study, we observed that presence of any fluid on OCT after 12 monthly injections is more prevalent in the bevacizumab treated patients (41%) than in the ranibizumab treated patients (30%). However, this is not reflected in different visual acuity outcomes. Up till now there is no explanation for this observation and more research is warranted.

That a possible more effective inhibition of leakage by ranibizumab is not always accompanied by a better visual acuity result could be due to the induction of progression of atrophy. This latter possibility is supported by recent analyses of the CATT and IVAN studies which indicated more rapid progression of atrophy in patients on monthly treatment than on PRN treatment.

Our study was designed specifically to compare the effects of maximal treatment of both VEGF- antagonists. Other studies ([3–6]) included also PRN treatment regimens which are often used in clinical practice. At present the two most commonly used variable treatment regiments are PRN, with monthly examinations, and a “treat and extend” strategy, where not only the number of injections but also the number of visits is reduced by extending the interval between visits based on clinical response[11]. Retreatment is often based on the presence of fluid on OCT rather than thickness values and the observation of more frequent fluid on OCT could hence have an influence on the number of injections when using PRN, leading to more injections for PRN treatment with bevacizumab. Retreatment criteria in these studies were based on a presence of fluid on OCT, haemorrhage, decrease in VA and presence of leakage on FA. In CATT slightly more injections were reported with bevacizumab (7.7 ±3.5) compared to ranibizumab (6.9 ± 3.0) and this was statistically significant. [6]. For IVAN these data are not published at the moment but the lowest count of ranibizumab injections reported is 6 compared to 7 for bevacizumab [3]. In MANTA there was no significant difference (p = 0.26) with slightly less injections in the ranibizumab group (5.8 ± 2.7) compared to bevacizumab (6.1 ± 2.8). [4]. In GEFAL, patients treated with bevacizumab received 6.8 (SD = 2.7)) injections compared to 6.5 (SD = 2.4) for patients treated with ranibizumab (p = 0.39).[5]

Bevacizumab and other anti-VEGF agents have serious side effects when used systemically for non-ocular indications, in particular in patients with an increased risk of stroke or other arterial thrombo-embolic events. [12] Bevacizumab and ranibizumab differ substantially in pharmacokinetics such as systemic half-life. It is possible therefore that they also differ in systemic side effects in ocular use. [13–15] However, to this day, no convincing differences in systemic adverse events have been shown. The BRAMD study did not have the statistical power to detect small but clinically meaningful differences in systemic safety. In future studies, our safety data will be pooled with the results of the other international trials comparing bevacizumab and ranibizumab for this purpose.

### Limitations

The follow-up time of patients in the BRAMD study is restricted to 12 months. We recognize that patients with AMD are often treated for years. It is highly conceivable however that results achieved during the first year of monthly treatment are predictive of future results. One year of intensive treatment should be sufficient to assess whether a patient will or will not continue to respond. Further, one year of treatment seemed sufficient to our public national funding agency as well to assess the non-inferiority of bevacizumab against ranibizumab.

In the IVAN trial, increased atrophy was observed in patients treated monthly with ranibizumab for 2 years, [7,15] The IVAN study group also reviewed this in the CATT 2 years results and confirmed their own observation. [16,17] This suggests that a trial with monthly injections might be safer if only conducted for one year.

The number of patients and the length of follow-up are not adequate to provide a full safety report on bevacizumab versus ranibizumab. Now that our study confirms the non-inferiority by visual acuity of bevacizumab against ranibizumab and market access of bevacizumab and ranibizumab in the Netherlands have been redefined as a result of it, we need further post-marketing surveillance to keep track of the safety in the long run.

We further recognize that we evaluated the efficacy of both drugs in a typically clinical trial setting, i.e. monthly injections while in every day practice most patients are treated on an as needed basis and we explicitly withhold ourselves from making statements about such treatment schedules.

What might be considered an inconsistency in our protocol is the definition of treatment failure on the one hand and the indication for becoming a switcher on the other hand. Patients are considered failures or losers to therapy once they lose 15 letters or more over the year. However, already when they were still ‘stabilizers’ with a loss of between 10 to 15 letters exclusive, a cross-over to the alternative drug was indicated. We applied an ad-hoc analysis to show the percentages of patients losing or gaining more than 10 rather than 15 letters with the latter reported in the results section of this paper. There were 57 patients gaining 10 letters or more in the bevacizumab group (35%) versus 61 (37%) in the ranibizumab group. For the losers this was 20 (12%) and 13 (8%) respectively (p = 0.41). This is consistent with the results above showing more losers in the bevacizumab group.

Three different SD-OCT systems were used. This could lead to some differences. In the MANTA conversion algorithms were used to deal with this problem. However we believe this is unnecessary as we analyzed the absolute differences between baseline and exit measurements, and as de Kinkelder et al. found a maximum difference in mean thickness between brands to be 7.7 micron (Topcon, Spectralis, Cirrus and RTVue) which is an insignificant difference [18].

Finally, we applied the last observation carried forward at the time of switching to the alternative drug. usually, this is not done in a intention-to-treat analysis, because the gain in vision after the switch is not accounted for. However, we did not want to run the risk of demonstrating non-inferiority of bevacizumab to ranibizumab because of patients switching therapies.

## Conclusion

The BRAMD study confirms that bevacizumab is non-inferior to ranibizumab in the treatment of exudative AMD. Our study did not result in new safety signals, but definitive results should come from safety data pooled over studies. Being much cheaper it is reasonable to suggest bevacizumab as the first choice of treatment in exudative AMD. However, in our study more patients obtained suboptimal results (more than 15 letters loss) in the bevacizumab than in the ranibizumab group. Therefore, for patients with an initial poor response to bevacizumab, ranibizumab may be useful as a second line treatment option.

## Supporting Information

S1 File The S1 file is the study protocol as it was approved by the medical ethical committee. (DOC)

S2 File The S2 file contains the Credit Roster. This provides an overview of the entire study group. It also lists what their specific role was within the study.
(DOC)

S3 File The S3 file is the consort work sheet. (DOC)

## Abbreviations

AMD: age-related macular degeneration
VEGF: Vascular Endothelial Growth factor
PRN: pro re nata
VA: distance visual acuity
OCT: optical coherence tomography
CNV: choroidal neovascularisation
BCVA: best corrected visual acuity
ETDRS: Early Treatment Diabetic Retinopathy Study
FA: fluorescein angiogram
SD- OCT: Spectral domain optical coherence tomography
CRT: central retinal thickness
ITT: intention to treat analysis
