# Supplementary material for: Comparing the Effectiveness of Bevacizumab to Ranibizumab in Patients with Exudative Age-Related Macular Degeneration. The BRAMD Study
Source: PLoS One. 2016 May 20;11(5):e0153052. doi: 10.1371/journal.pone.0153052 (PMC4874598; doi:10.1371/journal.pone.0153052)
Supplement: S1 File — (DOC) [file pone.0153052.s001.doc]

**Research Protocol**

**BRAMD Study**

**Comparing the effectiveness and costs of bevacizumab to ranibizumab in patients with exudative age-related macular degeneration**

**BRAMD trial**

**RO Schlingemann,**

**FD Verbraak,**

**MGW Dijkgraaf**

**R de Haan**

**Version 1**

**date 03-02-2008**

General Study characteristics

| Short title | Comparative study of bevacizumab and ranibizumab in AMD |
| --- | --- |
| Date | 7-12-2007 |
| Project leader | RO Schlingemann |
| Principal Investigators | G Dijkman  A Hooijmans  CB Hoyng  RO Schlingemann  H Vingerling |
| Steering Committee | MW Dijkgraaf  G Dijkman  R de Haan  A Hooijmans  CB Hoyng  RO Schlingemann (chairman)  FD Verbraak  H Vingerling |
| Sponsor | ZonMW |
| Independent physician | CP Nieuwendaal |

Protocol Signature Sheet

| **Name** | **Signature** | **Date** |
| --- | --- | --- |
| **Head of Department of …** |  |  |
| **Project leader** |  |  |

**List of abbreviations**

AE Adverse event

AMD Age-related macular degeneration

BCVA Best-corrected visual acuity

CNV Choroidal Neovascularization

CRC Central Reading Center

DSMB Data safety monitoring board

ETDRS Early Treatment Diabetic Retinopathy Study

ITT Intent-to-Treat

LOCF Last observation carried forward

OCT Optical coherence tomography

PDT Photodynamic therapy

RPE Retinal pigment epithelium

SAE Serious adverse event

VA Visual acuity

VEGF Vascular Endothelial Growth Factor

**Protocol Summary**

*Title*

Comparing the effectiveness and costs of bevacizumab to ranibizumab in patients with exudative age-related macular degeneration (BRAMD).

*Objective*

To compare the effectiveness and costs of 1.25 mg of bevacizumab to 0.5 mg ranibizumab, given as monthly intravitreal injections during one year.

*Study design*

This will be a randomized, controlled, double masked, clinical trial in 306 patients in five academic trial centres in The Netherlands.

*Study population*

Patients 60 years of age or higher with primary or recurrent sub- or juxtafoveal CNV secondary to AMD with a total area of CNV of < 12 disc areas and a best corrected visual acuity BCVA score between 78 and 20 letters (approximately 0,63-0,05) in the study eye

*Primary outcome*

Primary outcome measure will be the change in best-corrected visual acuity (BCVA) in the study eye from Baseline to Month 12.

*Secondary outcomes*

Secondary outcomes will be the proportions of patients with a gain of 15 letters or more and/or a BCVA of 20/40 or more at 12 months, the change in leakage and lesion size on fluoresce in angiography and change in foveal thickness by optical coherence tomography at 6 and 12 months, the number of adverse events in 12 months, and the costs and costs per quality adjusted life-year of the two treatments.

1. **Introduction and rationale**

Age-related macular degeneration (AMD) is the main cause of untreatable blindness in the

Netherlands.(1,2) AMD occurs in elderly people and occurs in a dry form, in which gradually progressive atrophy of the macula leads to loss of vision, and in a rapidly developing exudative form. Exudative AMD is responsible for most cases of severe vision loss, and is characterized by neovascularization and scarring under the retina.(2) It is estimated that 4000 new eyes develop exudative AMD in The Netherlands each year.(3) Most patients with exudative AMD are treated by ophthalmologists specialized in Medical Retina. Until recently, treatment options were thermal laser, photodynamic therapy and intra-vitreal injections with pegaptanib (4-6), but their efficacy is limited. Despite these treatments most AMD patients with neovascularization develop bilateral end stage disease and suffer from severe visual impairment within five years.(7)

In January 2007, an anti-angiogenic agent, ranibizumab (Lucentis), has been introduced, which represents a breakthrough in the treatment of exudative AMD.(8,9) Ranibizumab is a Fab fragment of a humanized monoclonal antibody against vascular endothelial growth-A (VEGF), a major causal factor in exudative AMD.(10) In three large randomized clinical trials, ranibizumab was shown to stabilize vision in 92-96 % of patients, when admitted by monthly intravitreal injections for one or two years, compared to stable vision in only 52-62% of patients treated with sham injections or photodynamic therapy.(8,9,11)

The maximum effect of ranibizumab was observed at three months, after which the effect stabilized. Parallel to its effect on visual acuity, ranibizumab markedly decreased retinal thickness as measured by optical coherence tomography (OCT).

The US Food and Drug Administration gave approval for the treatment of exudative AMD with

monthly injections of ranibizumab 0.5 mg on June 30, 2006 (Genentech Press release, June 30 2006, *http://www.gene.com/gene/news/pressreleases/display.do?method=detail&id=9787*). European approval has also been granted and the drug is available in the Netherlands since January 2007. It is registered in Europe to be used as three monthly injections with additional injections when needed. As the medication alone will cost 1200 Euro per injection, and as each patient will need up to 14 injections, the predicted costs in The Netherlands will be in excess of 60 million Euros per year. This is likely to lead to delayed introduction and limited access to this treatment, causing unnecessary blindness from AMD.

Bevacizumab (Avastin), produced by the same pharmaceutical company, is the full length anti-VEGF-A antibody from which ranibizumab is derived.(12) Bevacizumab has been used off-label on a widespread scale by ophthalmologists in the US and Europe in the treatment of exudative AMD.(12-15) No randomized controlled studies, but more than ten case series have been published, describing a total of more than 900 patients treated with bevacizumab. These suggest that bevacizumab is as effective and safe as ranibizumab. (16-20) In the largest case series (12), bevacizumab was found to improve visual acuity, as defined by a halving of the visual angle, in 38.3% of 144 patients at three months follow up. If confirmed in a state of the art trial, this would be comparable to the effect of ranibizumab. In addition, bevacizumab markedly reduced retinal thickness on OCT, to a similar extent as reported for ranibizumab. (13) The other published case series and personal experience of the project group members support these findings. A list of all published studies on bevacizumab in AMD is provided in the appendix 1.

Approximately 40,000 patients have been treated worldwide with bevacizumab. However, conclusive evidence from randomized controlled trials (RCT) directly comparing bevacizumab and ranibizumab is lacking. Such a RCT is not likely to be funded by the pharmaceutical industry. Since the approval and registration of ranibizumab, it has become difficult to treat patients with bevacizumab in the absence of evidence from RCT's showing that it is as effective as ranibizumab.

The main safety concerns regarding ocular use of ranibizumab are associated with the injection procedure, which is identical for injections with bevacizumab. However, as systemic bevacizumab, when given to patients with metastatic colorectal cancer, has a significantly increased risk of hypertension and arterial thrombo-embolic events including stroke and myocardial infarction, there is a chance that the 1000-fold lower ocular dose could have similar side-effects in some patients. This has been studied to some extent in an international survey, which collected safety data on 7113 injections in 5228 patients (21). In this open label, retrospective and uncontrolled study, only 0.21 % of patients experienced mild increases in systemic blood pressure, and cerebrovascular events were noted in 0,07% (3 patients). Due to the design of the study, these surprisingly low figures, which are lower than the background risk in this age group, may not represent the true incidence of systemic side effects. A complete overview of studies on bevacizumab containing information on systemic and ocular safety is given in Appendix 1.

The costs of Bevacizumab are 20- to 40 fold lower than of ranibizumab, and it has been estimated that in the Netherlands alone, the costs of ranibizumab treatment are around 30-50 million Euros higher than treatment with bevacizumab.

The purpose of this study is to generate conclusive evidence as to the non-inferiority in effectiveness of bevacizumab in the treatment of exudative AMD, in comparison with ranibizumab. In addition, data will be collected on the safety and costs of these treatments in AMD patients. Monthly injections for 12 months will be compared.

**2. Objectives**

*2.1 Primary objective*

The primary objective is to demonstrate the non-inferiority of bevacizumab to ranibizumab in the treatment of patients with subfoveal CNV secondary to AMD as determined by the change in best-corrected visual acuity in the study eye from baseline to month 12.

2.2. Secondary objectives

- To determine the difference between ranibizumab and bevacizumab in their effect on lesion leakage, lesion size and retinal thickness as determined by FA and OCT at 12 months
- To determine the difference in number of dropouts in the two treatment arms before the final 12 months assessments
- To determine the difference in number of non-responders in the two treatment arms at the 4 month assessment
- To determine the difference in the occurrence of (serious) adverse events in the 12 months study period
- To determine the difference in costs (and costs per quality adjusted life-year) between the two treatment strategies over the 12 months treatment period

**3. Study design**

The BRAMD study is a multicenter, randomized, double-masked clinical trial.

**4. Patient population**

*4.1 Patient population*

All patients of > 60 years of age with vision-threatening choroidal neovascularization (CNV) secondary to AMD that may benefit from anti-VEGF treatment are potentially eligible for the study.

4.2 Screening Phase

Potentially eligible patients referred to the study centres are informed of the possibility to enter the study. Patients will be given at least one week to read the Patient Information and will receive a phone call to discuss the study and planning of the screening visit. After giving informed consent, during the screening visit, (day -14 to baseline), patients will be definitely assessed for study eligibility based on the inclusion and exclusion criteria by visual acuity measurements, ophthalmic exams, optical coherence tomography and fluoresce in angiography.

*4.3 Inclusion criteria*

- Patients 60 years of age or higher.
- Patients with primary or recurrent sub-, juxta- or extrafoveal CNV secondary to AMD, including those with RAP, that may benefit from anti-VEGF treatment in the opinion of the investigator .
- The total area of CNV (including both classic and occult components) encompassed within the lesion must be more or equal to 30% of the total lesion area.
- The total lesion area should be < 12 disc areas.
- A best corrected visual acuity (BCVA) score between 78 and 20 letters (approximately 0,63-0,05 Snellen equivalent) in the study eye.

*4.4 Exclusion criteria*

- Ocular treatment with anti-angiogenic drugs in the last 2 months or Triamcinolone in the last 6 months.
- Laser photocoagulation (juxtafoveal or extrafoveal) in the study eye within one month preceding Baseline.
- Patients with angioid streaks or precursors of CNV in either eye due to other causes, such as ocular histoplasmosis, trauma, or pathologic myopia.
- Spherical equivalent of refractive error in the study eye demonstrating more than – 8 dioptres of myopia.
- Cataract extraction within three months preceding Baseline.
- IOP >25 mm Hg
- Active intraocular inflammation in the study eye.
- Vitreous haemorrhage obscuring view of the posterior pole in the study eye.
- Presence of a retinal pigment epithelial tear involving the macula in the study eye.
- Subretinal haemorrhage in the study eye if the size of the haemorrhage is > 70% of the lesion
- Subfoveal fibrosis or atrophy in the study eye.
- History of hypersensitivity or allergy to fluorescein.
- Inability to obtain fundus photographs, fluorescein angiograms or OCT’s of sufficient quality to be analyzed and graded by the Central Reading Centre.
- Systemic disease with a life expectancy shorter than the duration of the study.
- Inability to adhere to the protocol with regard to injection and follow-up visits.
- Legally incompetent adult
- Refusal to give written informed consent

*4.5 Sample size calculation*

As the measure of effectiveness, the mean BCVA score at month 12 in the ranibizumab group will be compared with the mean score in the bevacizumab group, corrected for baseline differences. Differences in the BCVA change scores from baseline will be tested statistically for non-inferiority. Starting from a common standard deviation of the change in BCVA score of 14 letters in both groups, and assuming an improvement from baseline of 9 letters in both the ranibizumab group and the bevacizumab group, a sample size of 306 patients (153 in each group) will have an 80% power of demonstrating non-inferiority by excluding a difference of 4 letters or more, using a one-sided t-test and a significance level of 0.05.

**5. Treatment of patients**

*5.1 Investigational treatments*

After informed consent and baseline assessment, the included patient is randomized to receive either:

- 1.25 mg bevacizumab, or
- 0.5 mg ranibizumab.

Both investigational treatments will be administered by monthly intravitreal injection for one year.

*5.2 Injection procedure (ranibizumab and bevacizumab)*

Ranibizumab and Bevacizumab are reconstituted and supplied by the hospital pharmacy in masked injection syringes containing a sterile solution of 0.3 ml of either 10 mg/ml of ranibizumab or 25 mg/ml bevacizumab. Of each compound, 0.05 ml is injected into the eye.

Patients will receive antimicrobials (e.g., Tobradex) three times daily for one day prior to treatment. Prior to the injection the peri-ocular skin and eyelid of the study eye are swapped with 10% povidone iodine swabs and an eyelid speculum is inserted. The syringe is inserted through an area 3.5–4.0 mm posterior to the limbus, avoiding the horizontal meridian and aiming toward the centre of the globe. The injection volume should be delivered slowly. The patient will be instructed to self-administer antimicrobial drops three times daily for three days following each injection. See appendix 1 for details.

To minimize the risk of patient delay in case of endophthalmitis or other ocular adverse events, patients are instructed to call the Department of Ophthalmology of their treating hospital if any ocular symptoms develop after the injection.

*5.3. Use of co-medication*

There are no restrictions as to co-medication.

*5.4 Use of escape medication*

Not applicable.

# 6. Investigational medical products

*6.1 Name and description of investigational products and product characteristics*

Ranibizumab and Bevacizumab are registered drugs. See appendix 3a and 3b for details.

## *6.2 Summary of known and potential risks and benefits*

See appendix 3a and 3b. For use of Bevacizumab in the eye, no extensive safety data are available. In the available case series, no drug- or injection related adverse events (or incidences) were noted other than observed for Ranibizumab (See Appendix 1).

## *6.3 Description of route of administration*

Ranibizumab and Bevacizumab are applied by intra-vitreal injection; for details see paragraph 5.1. and Appendix 2.

## *6.4 Dosages of investigational medical products.*

Ranibizumab and Bevacizumab are reconstituted and supplied by the hospital pharmacy in labelled masked injection syringes containing a sterile solution of 0.3 ml of either 10 mg/ml of Ranibizumab or 25 mg/ml bevacizumab. Of each compound, 0.05 ml is injected into the eye.

## *6.5. Preparation and labelling of Investigational Medicinal Product*

Reconstitution and labelling of the investigational medicinal products will be done by the Pharmacy Department, Erasmus MC, according to the GMP guidelines.

## *6.6 Drug accountability*

The shipment, receipt, disposition, return and destruction of the investigational medicinal products, when applicable, will be performed according to GDP guidelines.

**7. Methods**

*7.1 Study endpoints*

*7.1.1 Primary outcome*

*The primary outcome is* the change in best-corrected visual acuity (BCVA) in the study eye from Baseline to Month 12 assessed with ETDRS-like VA charts at an initial distance of four meter.

*7.1.2 Secondary outcomes*

-the proportion of patients with a loss of BVCA less than 15 letters from Baseline at 12 months (responders)

-the proportion of patients with a loss or gain of BVCA less than 15 letters from Baseline at 12 months (stabilizers)

-the proportion of patients with 15 letters loss or more of BCVA from Baseline at 12 months (losers)

-the proportion of patients with 15 letters gain or more of BCVA from Baseline at 12 months (gainers)

-the incidence of fluorescein leakage at 4 and 12 months as well as the change in total area of CNV, total area of leakage from CNV, and total lesion area from baseline at 12 months as determined by the reading centre

-absolute and percent change in retinal thickness, as measured by optical coherence tomography (OCT) at 4 and 12 months as determined by the reading centre

-proportion of dropouts before the final 12 months assessments

-proportion of non-responders at the 4 month assessment

-the occurrence of (serious) adverse events during the 12 months of the study.

-costs of the two treatments (see ECONOMIC EVALUATION)

## *7.2. Randomisation, blinding and treatment allocation*

Included patients are randomized to receive either bevacizumab or ranibizumab. Patient, treating physician, and evaluating investigator staff are blinded for treatment allocation. The randomization procedure will be computer- and web based, using permuted blocks and stratified by centre, BCVA of the study eye (52 letters or less versus 53 letters or more) , and BCVA of the non-study eye (52 letters or less versus 53 letters or more). Randomization will be made available by the AMC Clinical Research Unit.

*7.3 Study procedures*

At the baseline visit, the patient will sign the Informed Consent form, and the Medical and Ophthalmic History will be taken. Within 14 days after randomization the patient receives the first intravitreal injection of the study drug.

Investigations and measurements of the BRAMD trial are carried out according to the following diagram.

Study Flow Chart

| Assessment/  procedure | Phase |  | Treatment | | | | | | | | | | | | |
| --- | --- | --- | --- | --- | --- | --- | --- | --- | --- | --- | --- | --- | --- | --- | --- |
|  | Visit | 1 | 2 | 3 | 4 | 5 | 6 | 7 | 8 | 9 | 10 | 11 | 12 | 13 | 14 |
|  | Month | 0 | | 1 | 2 | 3 | 4 | 5 | 6 | 7 | 8 | 9 | 10 | 11 | 12 |
| Check inclusion/exclusion criteria |  | X |  |  |  |  |  |  |  |  |  |  |  |  |  |
| Informed consent |  | X |  |  |  |  |  |  |  |  |  |  |  |  |  |
| Medical History |  | X |  |  |  |  |  |  |  |  |  |  |  |  |  |
| Vital Signs |  | X |  | X | X | X | X | X | X | X | X | X | X | X | X |
| Check concomitant medications |  | X |  | X | X | X | X | X | X | X | X | X | X | X | X |
| Check adverse events |  |  |  | X | X | X | X | X | X | X | X | X | X | X | X |
| Drug administration |  |  | X | X | X | X | X | X | X | X | X | X | X | X |  |
| BCVA |  | X | X | X | X | X | X | X | X | X | X | X | X | X | X |
| Ophthalmic exam |  | X | X |  |  |  | X |  |  |  |  |  |  |  | X |
| Fluorescein angiography |  | X |  |  |  |  | X |  |  |  |  |  |  |  | X |
| Optical coherence tomography |  | X | X | X | X | X | X | X | X | X | X | X | X | X | X |
| Health care resource use questionnaire |  |  | X |  |  | X |  |  | X |  |  | X |  |  | X |

The following assessments will be performed at baseline and at the monthly visits, as indicated in the Study flow chart. During each visit, vital signs (pulse and blood pressure), concomitant medication and adverse events will be recorded. BCVA will be assessed and an OCT examination will be performed prior to the intravitreal injection. The interval between visits is 30 days, ±7 days to allow for flexibility in scheduling. Patients will be contacted by site personnel by telephone 2 days (± 1 day) after each study treatment as a safety follow-up. Patients will be explicitly asked whether they have taken the prescribed self-administered post-injection antimicrobial medications. At baseline, and at 4 and 12 months, an ophthalmic exam and fluorescein angiography will be performed. At baseline and at 3, 6, 9 and 12 months patients will be asked to complete a short, 16-item questionnaire on health status (EQ-5D), health care resource utilization, and out-of-pocket expenses (shortened Health and Labour questionnaire).

*7.3.1 Measurement of best corrected visual acuity*

Visual acuity (VA) will be assessed of both eyes at each study visit using best correction determined from protocol refraction. VA measurements will be taken in a sitting position using ETDRS – like visual acuity testing charts at an initial testing distance of 4 meters. Certification of the assessment procedures and examiners will occur prior to any evaluation of study patients.

*7.3.2 Ophthalmic exam*

The ophthalmic exam will include tonometry, eye motility and eyelid inspection, slitlamp anterior segment assessment and vitreous and posterior segment assessment with biomicroscopy.

*7.3.3 Fluorescein angiography*

Fluorescein angiography will be conducted in conjunction with colour fundus photography at Screening and at Months 4, 12. Investigators will use digital fluorescein angiograms to determine presence or absence of CNV and CNV lesion subtype (occult, classic, mixed or RAP) secondary to AMD at the baseline assessment. The following angiographic and photographic procedures are to be followed. After pupil dilation and prior to fluorescein dye injection, 35 degree red-free and colour stereoscopic pairs of photographs will be taken of the maculae of both eyes. Next, 5 ml of fluorescein 10% dye will be injected IV into the antecubital vein. Photographs of the study eye will be taken during the early transit phase from 15 to 45 seconds, at 60 to 90 seconds, and at 5 and 10 minutes after completion of the fluorescein dye injection. Photographs that are taken of the macula of the study eye during the fluorescein transit will typically include 1 stereoscopic pair at 3-5 minutes. Photographs of the macula of the fellow eye will be taken at 2 and 10 minutes after fluorescein dye injection. If the lesion is in a location that would be distorted or extends beyond the macular photographs, an additional photograph centered on the lesion should be taken during or following the transit phase. For both eyes, baseline and post-treatment assessment colour photographs and fluorescein angiograms will be sent to the CRC. A copy of the photographs and angiograms will be retained within the source documents. Photographers, systems and software will be certified prior to any evaluation of study patients.

*7.3.4 Optical coherence tomography*

Optical Coherence Tomography (OCT) will be performed of both eyes at Screening, and monthly from Month 1 through Month 12. OCT evaluations should be performed prior to study drug administration. These assessments will be performed by trained personnel at each site. OCT imaging will be performed using the Zeiss Humphrey System Model 2000 (or later) with version A6.1 (or more recent) software running under a Microsoft Windows environment. The ability to transfer images to digital video disc is required. Analysis of the OCT images will be performed by a CRC which will provide training materials. OCT operators, systems and software will be certified prior to any evaluation of study patients. To fully evaluate retinal details, the effect of the treatment on the CNV, and to monitor the effects of treatment on the retina, the following OCT scan patterns will also be evaluated by the CRC at Baseline/Visit 2 and all subsequent visits: 1. Fast macular thickness map with Default settings 2. Linear Cross Hair through the fovea with Scan length = 6mm (horizontal B-scan) 3. Linear Cross Hair through the fovea with Scan length = 6mm (vertical B scan) 4. Linear scan, length of 7 mm, from temporal margin of optic disc, with an angle of 355 degrees in the left eye, and with an angle of 5 degrees in right eye

*7.3.5 Central Reading Centre.*

An independent review of fundus photography, fluorescein angiography and OCT images will be

performed at the Central Reading Centre (CRC). The review teams will consist of 3

ophthalmologists experienced in clinical trials. The members are blinded for a patient’s treatment allocation. Certification of the assessment procedures and assessors will occur prior to any evaluation of study patients. The CRC will review fundus photography and fluorescein angiography to provide an objective assessment of, classification of lesion, lesion area, area of CNV, and lesion leakiness. The CRC will also review all OCT images to provide an objective, masked assessment of retinal thickness. and signs of active leakage.

Decisions for success or failure as described in 7.5 are at the discretion of the treating ophthalmologist.

*7.4 Withdrawal of individual subjects*

Patients can leave the study at any time for any reason if they wish to do so without any consequences. At time of early termination both the primary and secondary outcome parameters of the patient will be assessed and be regarded as the study outcome (last observation carried forward).

*7.5 Premature stopping of patient’s experimental treatment and cross-over to the ‘other’ study drug.*

At 4 months and at visits thereafter, the response to the treatment will be evaluated by the principal investigator. In case of a clearly inadequate response to the treatment, the patient is offered the possibility to stop receiving the initially assigned study drug and be treated for the next 4 months with the study drug of the other arm of the study.

A ‘non-response’ is defined as:

-a drop in BCVA of > 10 letters compared to baseline

AND

-clear evidence of active choroidal neovascularization, based on one of the following signs of CNV activity or leakage by qualitative OCT and fluorescein angiography assessment:

- at least two of the following signs of leakage on OCT:

-foveal thickening > 300 microns

-intraretinal cysts

-subretinal fluid

OR

-definite leakage of the CNV lesion on FA

When a patient stops receiving the initially assigned study drug due to non-response, a re-evaluation of the response to the ‘other’ medication will be carried out based on OCT and FA at 4 months after cross-over. When signs of leakage on OCT or FA have increased after the switch, the patients are offered to stop receiving the secondarily assigned drug and switch back to the originally assigned drug.

When a patient discontinues receiving the initially assigned study drug due to non-response, the assessments at 4 months will be regarded as the study outcome (last observation carried forward) regarding the drug’s effectiveness. All patients and study personnel will remain masked as to the treatment allocation. In addition, ‘cross-over’ patients will be monitored until the end of the study in order to include the health and costs consequences of premature termination of the initially assigned study drug in the economic evaluation.

*7.6 Premature termination of the study*

A Data Safety Monitoring Board will be installed and can recommend the Steering Committee of the BRAMD trial to terminate the study before completion (see further the paragraphs 8.6 and 9.2) depending on the level of discrepancy between both study arms in (i) incidences of (serious) adverse events and (ii) numbers of patients who terminate the initially assigned drug prematurely. Given the strict non-response definition, the latter argument is regarded as an indicator of potential harm.

# 8. Safety reporting

## *8.1 Section 10 WMO event*

In accordance to section 10, subsection 1, of the WMO, the investigator will inform the subjects and the DSMB if anything occurs, that implies that the disadvantages of participation are significantly greater than was foreseen. The study will be suspended pending further review by the DSMB, except insofar as suspension would jeopardise the subjects’ health. All subjects are informed and kept up to date by the investigator.

## *8.2 Adverse and serious adverse events*

Adverse events are defined as any undesirable experience occurring to a subject during a clinical trial, whether or not considered related to the investigational drug(s). All adverse events reported spontaneously by the subject or observed by the investiga­tor or his staff will be recorded.

A serious adverse event is any untoward medical occurrence or effect that at any dose:

- results in death;
- is life threatening (at the time of the event);
- requires hospitalisation or prolongation of existing inpatients’ hospitalisation;
- results in persistent or significant disability or incapacity;
- is a congenital anomaly or birth defect;
- is a new event of the trial likely to affect the safety of the subjects, such as an unexpected outcome of an adverse reaction, lack of efficacy of an IMP used for the treatment of a life threatening disease, major safety finding from a newly completed animal study, etc.

All SAEs will be reported to the DSMB and central METC, according to their requirements.

### *8.3 Suspected unexpected serious adverse reactions (SUSAR)*

Adverse reactions are all untoward and unintended responses to the investigational product(s) related to any dose administered. Unexpected adverse reactions are adverse reactions, of which the nature, or severity, is not consistent with the applicable product information i.e. the summary of the product characteristics as described in paragraph 6.

The investigator will report all SUSARs expedited to the DSMB and central METC, including SUSARs that have arisen in other clinical trials with the same medicinal product(s), and that could have consequences for the safety of the subjects involved in the BRAMD-Trial.

The expedited reporting will occur within 15 days after the investigator has first received information on the adverse reactions. For fatal or life-threatening cases the term will be maximal 7 days for a preliminary report with another 8 days for completion of the report.

The remaining SUSARs are recorded in an overview list (line listing) that will be submitted once every half year to the METC by the DSMB. This line listing provides an overview of all SUSARs from the study medicine, accompanied by a brief report highlighting the main points of concern.

The investigator will report all SUSARs expedited to the DSMB, METC, competent national authorities[[1]](#footnote-2) and the Data Safety Monitoring Board.

### *8.4 Annual safety report*

In addition to the expedited reporting of SUSARs, the investigator will submit, once a year throughout the clinical trial, a safety report to the central METC, and the competent authorities.[[2]](#footnote-3)

This safety report consists of:

- a list of all suspected (unexpected or expected) serious adverse reactions, along with an aggregated summary table of all reported serious adverse reactions, ordered by organ system;
- a report concerning the safety of the subjects, consisting of a complete safety analysis and an evaluation of the balance between the efficacy and the harmfulness of the treatment

## *8.5 Follow-up of adverse events*

All adverse events will be followed until they have abated, or until a stable situation has been reached. Depending on the event, follow up may require additional tests or medical procedures as indicated, and/or referral to the general physician or a medical specialist.

## *8.6 Data Safety Monitoring Board*

The Data Safety Monitoring Board (DSMB) is an independent committee of trial experts who will focus on safety monitoring. The DSMB consists of three members: 2 ophthalmologists and 1 epidemiologist. The DSMB will perform ongoing safety surveillances, especially with regard to the occurrence of serious adverse events in terms of arterial thrombo-embolic events. The investigator will report the occurrences of these events to the chairman on a weekly basis. In addition, the investigator reports the numbers of patients who do not respond to their initially assigned study drug. The DSMB may perform an interim analysis, upon the suspicion of one of the study drugs being more harmful than the other. The Steering Committee and the DSMB will agree on the approach to early termination (stopping rules) and the statistical methods used for the interim analysis beforehand. If the DSMB decides to perform an interim analysis, it will be done by an independent statistician of the Clinical Research Unit of the AMC, who has no responsibility for the management of the trial. The DSMB can recommend the Steering Committee of the BRAMD trial to early terminate the study when there is clear and substantial evidence of benefit (or less harm) of one study drug over the other. Clear and substantial benefit (or less harm) should be based on findings that are truly compelling while the risk of a false positive conclusion (type I error) is acceptable low.

**9. Statistical analysis**

According to the intention-to-treat principle all randomized patients will be included in the final analyses.

The primary outcome measure will be the change in best-corrected visual acuity (BCVA) from Baseline to Month 12 as assessed with ETDRS-like VA charts. When visual acuity is measured in this manner, a 15 letter gain means a doubling of the visual acuity, and a 15 letter loss means that acuity is halved. The ANCHOR and MARINA trials used a different primary outcome measure, namely the proportion of patients that lost less than 15 letters (‘responders’). In these trials, many patients treated with ranibizumab were found to gain letters instead of experiencing loss of acuity, and for that reason, all new ranibizumab trials running at present use the change in BCVA after one year as the primary outcome measure.

In the MARINA trial, the average BCVA score increased with 7.2 letters in the ranibizumab group versus a decrease of on average 10.5 letters in the placebo group at one year. In the ANCHOR study, mean changes were +11.3 letters in the group treated with ranibizumab group versus -9.5 in de placebo group (data based on Genentech press releases at http://www.gene.com/gene/news/pressreleases/display.do?method=detail&id=9787).

As the measure of effectiveness, the mean BCVA score at month 12 in the ranibizumab group will be compared with the mean score in the bevacizumab group, corrected for baseline differences. Differences in the BCVA change scores from baseline will be tested statistically for non-inferiority, using a one-sided t-test. Starting from a common standard deviation of the change in BCVA score of 14 letters in both groups, and assuming an improvement from baseline of 9 letters in both the ranibizumab group and the bevacizumab group, a sample size of 306 patients (153 in each group) will have an 80% power of demonstrating non-inferiority by excluding a difference of 4 letters or more, using a one-sided t-test and a significance level of 0.05. The standard deviation is based on observations in previous trials with ranibizumab. The mean improvement of 9 letters is the average of the changes observed in the placebo-controlled trials discussed earlier. The margin of non-inferiority is equivalent to half this average improvement. The analyses will be performed intention-to-treat based.

**9.1 Economic evaluation**

If the non-inferiority of bevacizumab to ranibizumab can be demonstrated, then the economic evaluation will be performed as a cost-minimization analysis from a societal perspective. If bevacizumab turns out to be inferior, then the question arises whether the health losses are in reasonable balance with the expected cost savings. In that situation a cost-utility analysis will be performed with the cost per quality-adjusted life-year as outcome parameter. The analysis will be based on (i) the observed cost and visual acuity data, and (ii) available and upcoming literature on health utility associated with different levels of visual acuity. (22,23) If a cost-utility analysis seems opportune, sensitivity analyses will be done to study the robustness of using patient-based preferences instead of general population based preferences in order to derive health utilities. The latter ones will be reported as the main outcome.

Costs will include the direct medical costs of diagnosis and treatment restricted to (potential) vision loss, including the use of visual aids. Only the medical costs attributable to loss of vision or the prevention thereof will be included in this population with a high risk of co-morbid conditions. Costs will be estimated as the product-sum of the volumes of resources used and their respective unit costs. The cost items will include visits to the health care providers (e.g. ophthalmologist, optometrists, and general practitioners), medication use, and ophthalmic equipment for imaging (Digital Imaging Systems, Fundus Camera's, Optical Coherence Tomography) and operating theatres. Patient-related costs will include the costs of health-related travel and over-the-counter medication. Within this aged population data on loss of productivity will not be collected. The use of resources will be documented in the case record forms and by an additional questionnaire to be completed at baseline and at 3-monthly intervals by the study participants. Unit costing will be based on the national guideline on costing in health care research, (29) supplemented by mean local unit costing data from participating reference centres. The base year for costing will be 2008. Unit costs will be price-indexed when originating from other calendar years using general yearly price-indices. (24)

The time horizon of the economic evaluation will be one full year. No discounting of costs (and effects) will be performed.

**9.2 Predefined subgroup-analysis**

With respect to the primary outcome a predefined subgroup-analysis will be performed based on lesion subtypes.

**10. Ethical considerations**

## *10. 1 Regulation statement*

The BRAMD trial will be conducted according to the principles of the Declaration of Helsinki (version of 2004) and in accordance with the Medical Research Involving Human Subjects Act (WMO) and other guidelines, regulations and Acts. Data management, monitoring and reporting of the study will be performed in accordance with the ICH GCP guidelines.

## *10. 2 Recruitment and consent*

If inclusion and exclusion criteria are met, patients will be asked to participate. After patients have been written informed about all aspects of the study, they confirm their participation by filling out and signing an informed consent form.

The following basic elements are documen­ted in the patient information letter:

- A statement that the study involves research;
- A full and fair explanation of the procedures to be followed, identifying which of them are experimental;
- A full explanation of the nature, expected duration, and purpose of the study;
- A description of any reasonable foreseeable risks or discomfort to the patient;
- A description of any benefits which may reasonably be expected;
- A statement that participation is voluntary, that refusal to participate will involve no penalty or loss of bene­fits to which the patient is other­wise entitled, and that the patient may discontinue participation at any time without penalty or loss of benefits, in which case the patient will receive standard or another treatment with the same degree of care.

The patient information letter and informed consent form are provided in Appendix …

## *10.3 Objection by minors or incapacitated subjects*

Minors and legally incompetent adults are excluded from the study.

## *10.4 Compensation for injury*

The AMC Medical Research BV has insurance which is in accordance with the legal requirements in the Netherlands (Article 7 WMO and the Measure regarding Compulsory Insurance for Clinical Research in Humans of 23rd June 2003). This insurance provides cover for damage to research subjects through injury or death caused by the study.

1. € 450.000,-- (i.e. four hundred and fifty thousand Euro) for death or injury for each subject who participates in the Research;
2. € 3.500.000,-- (i.e. three million five hundred thousand Euro) for death or injury for all subjects who participate in the Research;
3. € 5.000.000,-- (i.e. five million Euro) for the total damage incurred by the organisation for all damage disclosed by scientific research for the AMC as sponsor ('verrichter') in the meaning of said Act in each year of insurance coverage.

The insurance applies to the damage that becomes apparent during the study or within 4 years after the end of the study.

*10.5 Incentives*

Enrolled patients will not receive any special incentives, compensation or treatment through participation in this study.

# 11. Administrative aspects and publication

# *11. 1 Handling and storage of data*

The investigator will set up a Trial Master File at the beginning of the study. The list of essential documents will be in accordance with the GCP-guidelines. The essential documents that make up the file will be stored in a secure but accessible manner. All essential documents will be legible and accurate. The participating centres will keep copies of relevant documents, including essential center-specific documents. A copy of the fundus photographs, fluorescein angiograms, OCT images, and visual field tests will be retained with the source documents at each investigative site.

Randomisation software will be made available by the AMC Clinical Research Unit.

After the end of the study all essential documents pertaining to the conduct of the study, (e.g., screening forms, digital Case Record Forms (CRFs), patient files, originals of test result reports, corres­ponden­ce, records of informed consent, etc) will be archived by the investiga­tor for a period of 15 years in accordance with the standard operating procedure of the AMC.

## *11.2 Handling and storage of documents*

For each randomized patient a digital Case Record Form (CRF) will be com­pleted. The CRF consists of a sequential set of in­structions with provision for data recording.

All randomized patients are identified by a Patient Identifi­cation Number (PIN) in combination with a center number. Trial personnel will not pass names outside the local hospital. The investigator will insure that patients' anonymity is maintained. On screening forms, digital CRFs or other documents submitted to the coordinating center, patients will not be identified by their names but by a PIN in combination with a center number. The subject identification code list will safeguarded by the investigator.

Central data management will be performed in Oracle Clinical by technicians and data managers of the AMC Clinic Research Unit. Internet-based remote data capture will be used for entering, managing and validating data from the investigative sites. Oracle Clinical was designed to meet industry regulations, including:

- FDA 21CFR Part 11 Rule (March 20, 1997),
- ICH; Good Clinical Practice: Consolidated Guideline (May 9, 1997)
- FDA Guidance for Industry “Computerized Systems Used In Clinical Trials” (May 10, 1999)

## *11. 3 Amendments*

Amendments are changes made to the research after a favourable opinion by the central METC has been given.

A ‘substantial amendment’ is defined as an amendment to the terms of the METC application, or to the protocol or any other supporting documentation, that is likely to affect to a significant degree:

- the safety or physical or mental integrity of the subjects of the trial;
- the scientific value of the trial;
- the conduct or management of the trial; or
- the quality or safety of any intervention used in the trial.

All substantial amendments will be notified to the METC and to the competent authority.[[3]](#footnote-4)

Non-substantial amendments will not be notified to the accredited METC and the competent authority, but will be recorded and filed in the Trial Master File by the investigator.

## *11. 4 Trial registration*

The study protocol will be admitted to the 'Nederlands Trial Register' according to their guidelines.

## *11. 5 Annual progress report*

The investigator will submit a summary of the progress of the trial to the accredited METC once a year. Information will be provided on the date of inclusion of the first subject, numbers of subjects included and numbers of subjects that have completed the trial, serious adverse events/ serious adverse reactions, other problems, and amendments.

*11. 6 End of study report*

The investigator will notify the accredited METC and the competent authority of the end of the study within a period of 90 days. The end of the study is defined as the moment the last patient reaches his primary outcome at three months follow-up.

In case the study is ended prematurely, the investigator will notify the accredited METC and the competent authority within 15 days, including the reasons for the premature termination.

Within one year after the end of the study, the investigator will submit a final study report with the results of the study, including any publications/abstracts of the study, to the accredited METC and the Competent Authority.

## *11. 7 Public disclosure and publication policy*

The Steering Committee is the main policy and decision making committee of the BRAMD study and has the final responsibility for the scientific conduct of the study. It is composed of representatives of the investigators. The specific tasks of the Steering Committee are to approve study reports and papers for publication...

**References**

1 Vingerling JR, Dielemans I, Hofman A, et al., The prevalence of age-related maculopathy in the Rotterdam Study, Ophthalmology, 1995, 102, 205-210.

2 Schlingemann RO, Role of growth factors and the wound healing response in age-related macular degeneration, Graefes Arch Clin Exp Ophthalmol, 2004, 242, 91-101.

3 van Leeuwen R, Klaver CC, Vingerling JR, Hofman A and de Jong PT, The risk and natural course of age-related maculopathy: follow-up at 6 1/2 years in the Rotterdam study, Arch Ophthalmol, 2003, 121, 519-526.

4 Schmidt-Erfurth U, Miller JW, Sickenberg M, et al., Photodynamic therapy with verteporfin for choroidal neovascularization caused by age-related macular degeneration: results of retreatments in a phase 1 and 2 study, Arch Ophthalmol, 1999, 117, 1177-1187.

5 Macular Photocoagulation Study Group, Argon laser photocoagulation for neovascular maculopathy. Three-year results from randomized clinical trials. Arch Ophthalmol, 1986, 104, 694-701.

6 VEGF Inhibition Study in Ocular Neovascularization Clinical Trial Group. Pegaptanib for neovascular age-related macular degeneration.New England Journal of Medicine 2004; 351: 2805-2816.

7 Risk factors for choroidal neovascularization in the second eye of patients with juxtafoveal or

subfoveal choroidal neovascularization secondary to age-related macular degeneration. Macular

Photocoagulation Study Group, Arch Ophthalmol, 1997, 115, 741-747.

8 Rosenfeld PJ, Brown DM, Heier JS, Boyer DS, Kaiser PK, Chung CY, Kim RY; MARINA Study Group. Ranibizumab for neovascular age-related macular degeneration. N Engl J Med. 2006;355:1419-31 4 Spaide RF, Laud K, Fine HF, et al., Intravitreal bevacizumab treatment of choroidal neovascularization secondary to age-related macular degeneration, Retina, 2006, 26, 383-390.

9 Brown DM, Kaiser PK, Michels M, Soubrane G, Heier JS, Kim RY, Sy JP, Schneider S; ANCHOR Study Group. Ranibizumab versus verteporfin for neovascular age-related macular degeneration. N Engl J Med. 2006;355:1432-44.

10 Witmer AN, Vrensen GF, Van Noorden CJ and Schlingemann RO, Vascular endothelial growth factors and angiogenesis in eye disease, Prog Retin Eye Res, 2003, 22, 1-29.

11 Heier JS, Boyer DS, Ciulla TA, Ferrone PJ, Jumper JM, Gentile RC, Kotlovker D, Chung CY, Kim RY; FOCUS Study Group. Ranibizumab combined with verteporfin photodynamic therapy in neovascular age-related macular degeneration: year 1 results of the FOCUS Study.

Arch Ophthalmol. 2006;124:1532-42.

12 Bevacizumab. Anti-VEGF monoclonal antibody, avastin, rhumab-VEGF, Drugs R D, 2002, 3, 28-30.

13 Lazic R, Gabric N. Intravitreally administered bevacizumab (Avastin) in minimally classic and occult choroidal neovascularization secondary to age-related macular degeneration. Graefes Arch Clin Exp Ophthalmol. 2007;245:68-73

14 Rosenfeld PJ, Moshfeghi AA and Puliafito CA, OCT findings after an intravitreal injection of

bevacizumab (avastin) for neovascular age-related macular degeneration, Opht Surg Lasers Imag, 2005, 36, 331-335.

15 Avery RL, Pieramici DJ, Rabena MD, Castellarin AA, Nasir MA and Giust MJ, Intravitreal

bevacizumab (Avastin) for neovascular age-related macular degeneration, Ophthalmology, 2006, 113, 363-372 e365.

16 Manzano RP, Peyman GA, Khan P and Kivilcim M, Testing intravitreal toxicity of bevacizumab (Avastin), Retina, 2006, 26, 257-261.

17 Maturi RK, Bleau LA and Wilson DL, Electrophysiologic findings after intravitreal bevacizumab (Avastin) treatment, Retina, 2006, 26, 270-274.

18 Shahar J, Avery RL, Heilweil G, et al., Electrophysiologic and retinal penetration studies following intravitreal injection of bevacizumab (Avastin), Retina, 2006, 26, 262-269.

19 Kiss C, Michels S, Prager F, Weigert G, Geitzenauer W, Schmidt-Erfurth U. Evaluation of anterior chamber inflammatory activity in eyes treated with intravitreal bevacizumab. Retina. 2006;26:877-81.

20 Spandau UH, Jonas JB. Retinal pigment epithelium tear after intravitreal bevacizumab for exudative age-related macular degeneration. Am J Ophthalmol. 2006;142:1068-70.

21 14. Rich RM, Rosenfeld PJ, Puliafito CA, et al. Short-term safety and efficacy of intravitreal bevacizumab (Avastin) for neovascular age-related macular degeneration. Retina 2006;26(5):495-511.

22 Brown GC, Sharma S, Brown MM, Kistler J. Utility Values and Age-related macular Degeneration. Arch Ophthalmol 2000; 118(1):47-51

23 Espallargues M, Czoski-Murray CJ, Bansback NJ, Carlton J, Lewis GM, Hughes LA et al. The Impact of Age-Related Macular Degeneration on Health Status Utility Values. Invest Ophthalmol Vis Sci 2005; 46(11):4016-4023

24 Oostenbrink JB, Koopmanschap MA, Rutten FF. Standardisation of costs: the Dutch Manual for Costing in economic evaluations. Pharmacoeconomics 2002; 20(7):443-454.

**Appendix 1**

A systematic survey was carried out in the literature of articles containing safety information on ocular use of bevacizumab. We finally included sixteen articles on follow-up studies in patients who received Bevacizumab for exudative AMD for this review (table). ^1^ ^2 3 4 5 6 7 8 9 10 11 12 13 14 15 16^ A total of 947 patients were included in these studies. Two articles showed the results of the same study. ^12 13^ The article with the largest series of patients and longest follow-up period was included in this review. ^13^ In this study the effect of intravenous bevacizumab in AMD was studied. Two other studies included patients who had received intravenous bevacizumab. ^6 8^ The data of these three studies were included in the summary of the effect of intravenous injections.

Thirteen articles reported the change in VA after intravitreal Bevacizumab (see table). ^1 2 3 11 5 7 8 9 10 11 14 15^

**Reported adverse events of 16 studies on the effect of bevacizumab in exudative AMD.**

| **Treatment** | **Number of patients at follow-up; time at follow-up in weeks** | **Adverse events** |
| --- | --- | --- |
| Intravenous. 2-3 at two weeks interval. Additional therapy according to criteria | n=18; 24 weeks | No serious ocular or systemic adverse events. Increase in systolic and diastolic blood pressure. |
| Intravenous. 3 with 2 weeks in between | n=9 ; 12 weeks | No |
| Intravenous. 3 with 2 weeks in between | n=12; 12 weeks | No severe adverse events |
| Intravitreal, 1, 5 mg. No additional injections | n=66; 8 weeks (at maximum change) | Not reported |
| Intravitreal. 2.5 mg. No additional injections. | n=39; 4 weeks | No severe adverse effects were observed during follow-up period. A mild inflammatory response in the anterior chamber was the most common side effect observed in 8 eyes (20.5%), followed by subconjunctival hemorrhage in 3 patients |
| Intravitreal. 3 x 2, 5 mg with 4 weeks in between. No additional injections | n=17; 12 Weeks | No (ocular) complications. No blood pressure rise, no thrombo-embolic events. |
| Intravitreal.3 x 1 mg with 4 weeks in between. No additional injections | n=12; 12 weeks | No severe adverse events |
| Intravitreal 1, 25 mg. One injection. Additional injections according to criteria | n=30; 14 weeks | No serious ocular or systemic events were reported within 12 weeks after treatment. No significant elevation of blood pressure or of intraocular pressure, no endophthalmitis, no retinal toxicity, no intraocular inflammation or uveitis. |
| Intravitreal. 1.25 mg. One injection. Additional injections according to criteria | n=50; 8 weeks | No uveitis, endophthalmitis, ocular toxicity, thrombo-embolic events, no significant increase in blood pressure |
| Intravitreal. 1.25 mg. One injection. Additional injections according to criteria | n=80; 12 weeks (range 6-24) | RIP in 2 patients. 10 PVD. No inflammation, infection, thrombo-embolic event, or ocular toxicity. No cataract progression. |
| Intravitreal. 1.25 mg. One injection. Additional injections according to criteria | n=50; 12 weeks | No episodes of inflammation or vision loss immediately after the injection. No endophthalmitis, retinal detachment or lens damage. No thrombo-embolic events including: CVA, MI, TIA, peripheral vascular disease. |
| Intravitreal. 1, 25 mg. Three injections. Additional injections according to criteria | n=141; 12 weeks | At 1 month, two patients had mild vitritis, and one patient at 2 months, who had a history of recurrent uveitis. No endophthalmitis, increased intraocular pressure, retinal tear, or retinal detachment occurred. No other adverse events in 591 injections, but one patient had MI after third injection; one treated for TIA stopped anti-coagulants by himself and developed TIA. Another possible TIA, One death of MI (smoker with emphysema). |
| Intravitreal. 1, 25 mg. One injection Additional injections according to criteria | n=48; 24 weeks | No incidence of severe vision loss or adverse effect |
| Intravitreal. 1,25 mg or 1.5 mg. Additional injections according to criteria | n=80; 12 week | No intraocular inflammation and no thrombo-embolic events |
| Intravitreal. 1.25 mg. One injection. Additional injections according to criteria | n=102; 10 weeks | No adverse ocular or systemic events. |
| Intravitreal. 1.25 mg. One injection. Additional injections according to criteria | n=17; 18 weeks (mean follow-up) | Intravitreal bevacizumab was well tolerated in all patients. No patient developed uveitis, endophthalmitis, ocular toxicity or thrombo-embolic events. |

^16^ In two studies only one injection was given. ^1 9^ In ten studies 1.0, 1.25 or 1.5 mg intravitreal bevacizumab was given repeatedly. ^2 3 4 7 8 10 11 21 15^ ^16^

Adverse events were rare and severe adverse were not observed in the 908 patients included in these studies and the several thousands of injection applied to these patients. Although the reported thrombo-embolic events could have been caused by bevacizumab the time frame between injection and the event or the presence of additional risk factors make this less likely. ^15^ In one study the occurrence of a rip or rupture of the detached retinal pigment epithelium (RPE) was reported in two patients. ^11^ Such an event may also occur as a result of the natural course of AMD itself or with other therapies such as photodynamic therapy and ranibizumab. ^17^ ^18^

These results confirm the results of an internet surveillance program in which the follow-up for adverse events in 5228 patients and 7113 intravitreal injections with bevacizumab from 70 centres in twelve countries was reported. Adverse events reported were corneal abrasion, lens injury, endophthalmitis, retinal detachment, inflammation or uveitis, cataract progression, acute vision loss, central retinal artery occlusion, subretinal haemorrhage, retinal pigment epithelium tears, blood pressure elevation, transient ischemic attack, cerebrovascular accident and death. None of the adverse event rates exceeded 0.21%. ^19^ These results are also confirmed by a 12 month follow-up study of 1265 consecutive patients with several diagnoses, not only exudative AMD, who received 4303 intravitreal injections of bevacizumab. ^20^ Systemic adverse events occurred in eighteen patients (1.5%). These were: acute elevation of blood pressure (0.59%), cerebrovascular accidents (0.5%), myocardial infarction (0.4%), iliac artery aneurisms (0.17%) and toe amputation (0.17%) and deaths (0.4%). Ocular adverse events were bacterial endophthalmitis (0.16%), tractional retinal detachment (0.16%), uveitis (0.09%), rhegmatogenous retinal detachment (0.02%) and vitreous haemorrhage. Some of the adverse events may have been the results of the underlying disease.

References

1. Abraham Marin ML, Cortes Luna CF, Alvarez Rivera G, et al. Intravitreal bevacizumab therapy for neovascular age-related macular degeneration: a pilot study. Graefes Arch Clin Exp Ophthalmol 2006;245:651-5.

2. Aggio FB, Farah ME, Silva WC, Melo GB. Intravitreal bevacizumab for exudative age-related macular degeneration after multiple treatments. Graefes Arch Clin Exp Ophthalmol 2006;245:215-20.

3. Aisenbrey S, Ziemssen F, Volker M, et al. Intravitreal bevacizumab (Avastin) for occult choroidal neovascularization in age-related macular degeneration. Graefes Arch Clin Exp Ophthalmol 2006;245:941-8.

4. Avery RL, Pieramici DJ, Rabena MD, et al. Intravitreal bevacizumab (Avastin) for neovascular age-related macular degeneration. Ophthalmology 2006;113(3):363-72.e5.

5. Bashshur ZF, Bazarbachi A, Schakal A, et al. Intravitreal Bevacizumab for the Management of Choroidal Neovascularization in Age-related Macular Degeneration. American Journal of Ophthalmology 2006;142:1-9.

6. Bolz M, Michels S, Geitzenauer W, et al. Effect of systemic bevacizumab therapy on retinal pigment epithelial detachment. Br J Ophthalmol 2006(oct 18):10.1136/bjo.2006.102467.

7. Chen CY, Wong TY, Heriot WJ. Intravitreal Bevacizumab (Avastin) for Neovascular Age-related Macular Degeneration: A Short-term Study. Am J Ophthalmol 2007;143(3):510-2.

8. Geitzenauer W, Michels S, Prager F, et al. Early effects of systemic and intravitreal bevacizumab (AvastinR) therapy for neovascular age-related macular degeneration. Klinische Monatsblatter fur Augenheilkunde 2006;223(10):822-7.

9. Jonas JB, Libondi T, Ihloff AK, et al. Visual acuity change after intravitreal bevacizumab for exudative age-related macular degeneration in relation to subfoveal membrane type. Acta Ophthalmol Scand 2007:10.1111/j.600-0420.2007.00891.x.

10. Ladewig MS, Ziemssen F, Jaissle G, et al. Intravitreal bevacizumab for neovascular age-related macular degeneration. Ophthalmologe 2006;103(6):463-70.

11. Lazic R, Gabric N. Intravitreally administered bevacizumab (Avastin) in minimally classic and occult choroidal neovascularization secondary to age-related macular degeneration. Graefes archive for clinical and experimental ophthalmology 2007;245(1):68-73.

12. Michels S, Rosenfeld PJ, Puliafito CA, et al. Systemic bevacizumab (Avastin) therapy for neovascular age-related macular degeneration: Twelve-week results of an uncontrolled open-label clinical study. Ophthalmology 2005;112(6):1035-47.

13. Moshfeghi AA, Rosenfeld PJ, Puliafito CA, et al. Systemic bevacizumab (Avastin) therapy for neovascular age-related macular degeneration: twenty-four-week results of an uncontrolled open-label clinical study. Ophthalmology 2006;113(11):2002.e1-12.

14. Rich RM, Rosenfeld PJ, Puliafito CA, et al. Short-term safety and efficacy of intravitreal bevacizumab (Avastin) for neovascular age-related macular degeneration. Retina 2006;26(5):495-511.

15. Spaide RF, Laud K, Fine HF, et al. Intravitreal bevacizumab treatment of choroidal neovascularization secondary to age-related macular degeneration. Retina 2006;26(4):383-90.

16. Yoganathan P, Deramo VA, Lai JC, et al. Visual improvement following intravitreal bevacizumab (Avastin) in exudative age-related macular degeneration. Retina 2006;26(9):994-8.

17. Gelisken F, Inhoffen W, Partsch M, et al. Retinal pigment epithelial tear after photodynamic therapy for vhoroidal neovascularization. Am J Ophthalmol 2001;131:518-20.

18. Carvounis PE, Kopel AC, Benz MS. Retinal pigment epithelial tears following ranibizumab for exudative age-realted macualr degeneration. Am J Ophthalmol 2007;143:504-5.

19. Fung AE, Rosenfeld PJ, Reichel E. The International Intravitreal Bevacizumab Safety Survey: Using the internet to assess drug safety worldwide. British Journal of Ophthalmology 2006;90(11):1344-9.

20. Wu L, Martinez-Castellanos MA, Quiroz-Mercado H, et al. Twelve-moth safety of intravitreal injections of bevacizumab (Avastin): results of the Pan-American Collaborative Retina Study Group (Pacores). Graefes Arch Clin Exp Ophthalmol 2007;Aug 3; [Epub ahead of print].

**Appendix 2** Injection procedure (ranibizumab and bevacizumab)

Ranibizumab and bevacizumab are reconstituted and supplied by the hospital pharmacy in injection syringes containing a sterile solution of 0.3 ml of 10 mg/ml ranibizumab or 0.3 ml of 25 mg/ml bevacizumab, respectively. Of each compound, 0.05 ml is injected into the eye.

The following procedures will be implemented to minimize the risk of potential adverse events

associated with serial intravitreal injections. Aseptic technique will be observed by clinic staff involved in the injection tray assembly, anaesthetic preparation, and study drug preparation and administration. In addition to the procedures outlined below, added safety measures in adherence to specific institutional policies associated with intravitreal injections will be observed.

Patients will receive antimicrobials (e.g., ofloxacin ophthalmic) three times daily for three days prior to treatment. The following procedures will be conducted by the injecting physician. -The technician assembles the supplies and prepares a sterile field. Supplies include 10% povidone iodine swabs, sterile surgical gloves, 4×4 sterile pads, pack of sterile cotton-tipped applicators, eyelid speculum, sterile ophthalmic drape, 0.5% proparacaine hydrochloride, 5% povidone iodine ophthalmic solution, ophthalmic antimicrobial solution , and injection supplies. - Install 2 drops of 0.5% proparacaine hydrochloride into the study eye, followed by 2 drops of antimicrobial solution. – Disinfect the periocular skin and eyelid of the study eye in preparation for injection. Scrub the eyelid, lashes, and periorbital skin with 10% povidone iodine swabs, starting with the eyelid and lashes and continuing with the surrounding periocular skin. Make certain that the eyelid margins and lashes are swabbed, and proceed in a systematic fashion, from medial to temporal aspects. - The physician will glove, place sterile ophthalmic drape to isolate the field, and place the speculum underneath the eyelid of the study eye. - Install 2 drops of 5% povidone iodine ophthalmic solution in the study eye, making sure the drops cover the planned injection site on the conjunctiva. - Wait 90 seconds. - Saturate a sterile cotton-tipped applicator with 0.5% proparacaine hydrochloride drops and hold the swab against the planned intravitreal injection site for 10 seconds in preparation for the intravitreal injection. – The labelled syringe allocated to the patient containing the study medication is fitted it with a 30-gauge needle. Insert the syringe through an area 3.5–4.0 mm posterior to the limbus, avoiding the horizontal meridian and aiming toward the centre of the globe. The injection volume of 0.05 ml should be delivered slowly. The needle should then be removed slowly to ensure all drug solution is in the eye. The scleral site for subsequent intravitreal injections should be rotated. - Immediately following the injection, instil two drops of antimicrobial drops (e.g., ofloxacin

ophthalmic solution) in the study eye. -The patient will be instructed to self-administer antimicrobial drops four times daily for three days following each injection.

1. The Dutch central committee for research with humans, CCMO (De Centrale Commissie Mensgebonden Onderzoek ) and the committee that considers medication in The Netherlands, CBG (College ter Beoordeling van Geneesmiddelen). [↑](#footnote-ref-2)
2. The Dutch central committee for research with humans, CCMO (De Centrale Commissie Mensgebonden Onderzoek ) [↑](#footnote-ref-3)
3. The Dutch central committee for research with humans, CCMO (De Centrale Commissie Mensgebonden Onderzoek ) [↑](#footnote-ref-4)
