# Supplementary material for: Comparing the Effectiveness of Bevacizumab to Ranibizumab in Patients with Exudative Age-Related Macular Degeneration. The BRAMD Study
Source: PLoS One. 2016 May 20;11(5):e0153052. doi: 10.1371/journal.pone.0153052 (PMC4874598; doi:10.1371/journal.pone.0153052)
Supplement: S2 File — This provides an overview of the entire study group. It also lists what their specific role was within the study. (DOC) [file pone.0153052.s002.doc]

Credit Roster for the BRAMD

Clinical Centers (Ordered by Number of Patients enrolled)

Certified Roles at Clinical Centres: Clinic Coordinator (CC), Data Entry Staff (DE), Participating Ophthalmologist (O), Ophthalmic Photographer (OP); Optical Coherent Tomography Technician (OCT), Principal Investigator (PI), Refractionst (R), Visual Acuity Examiner (VA)

AMC: Reinier O. Schlingemann (PI), Frank D. Verbraak (O), Marie van Schooneveld (O), Monique Wezel (CC), Henk Stam (VA/R/OCT), Christa Jansen-Kok (VA/R/OCT/DE), Ans Althoff (VA/R/OCT) Douwe Bakker (CC/VA/R/OCT/DE), Annette van der Zee (DE), Dick De Vries (OP)

Erasmus MC: Johannes R. Vingerling (PI), Naus- Postema (O), De Roo Hertoge (O), C. Klaver (O), E. Kilic (O), Yvonne Noordzij (CC/DE/VA/R/OCT), Jeanette Noordzij (CC/DE/VA/R/OCT), Anjo Vermij (OCT/VA/R), Ada Hooghart (OCT/VA/R)

LUMC: Greetje Dijkman (PI), Ingrid Boesten (CC/DE/VA/R/OCT), C. Kiewiet de Jonge (VA/R/OCT), M. Kromhart- de Haas (VA/R/OCT), Cora Mollinger (VA/R), J.W. Zwaan (VA/R), Lou Brink (VA/R), Anneke Boolman (VA/R)

UMCG: Johanna Hooymans (PI), Nicole Kamminga (O), Angela Huiskamp (O), Postma (O) Marijke Meinen (CC/DE/R/VA), L. Uwantege (VA/R), H.R. Luurtsema (VA/R), J.F. Eisses (VA/R/OCT), Westra (OP),

UMCN: Carel B. Hoyng (PI), Agnes de Vries (CC), Elke Huntink (CC), Asha Kalisingh (CC), Liesbeth Hoeks (VA/R),Hans Hermans (VA/R), Angeline Rottelveel (VA/R/OCT), J. Weeda (VA/R/OP/OCT), Chantal Van Ast (OCT)

OMC Haarlem: Annemieke Coops (VA/R), R. Van Hierden (OCT/VA/R), Kees Corstanje (VA/R), Madelon Jansen (VA/R/OCT), Nikki van denBerg (VA/R/OCT), J.H.J. Klaver (O), M. H. van der Linden (O)

Zonnestraal Hilversum: Bianca van Leeuwen (VA/R/DE), Gerrie Dantuma (VA/R/OCT), Serge Koning (VA/R/OCT), Maurice van Baekel (OCT/VA/R), Mike Selders (VA/R/OCT), Martine Zandee (VA/R/OCT), Maaike Goudriaan (VA/R/OCT), Annemarieke Langen (VA/R/OCT), R.W.H van de Mortel (VA/R/OCT), A. Rulo, F. Gerbrandy, Antoine Tromp, Oscar Lopes Cardozo

Resource Centers

Chairman’s Office and Coordinating Center(Academic Medical Center, Amsterdam, the Netherlands): R.O. Schlingemann, MD Phd(Chair/PI); F.D Verbraak,

MD (Vice-Chair; Academic Medical Center, Amsterdam, the Netherlands)

M.G.W. Dijkgraaf (methodologist), R. De Haan (methodologist), A.H. Zwinderman, SCP (Biostatistician); A.M.E. Schauwvlieghe, MD (Medical Monitor); Selma Mehmedovic (Protocol Monitor); Jaqueline van Dalen (CRA), Irmgard Corten (Systems Analyst); Erik Veenstra (Financial Administrator); Jolanda Strubel (Database developer)

OCT Reading Center: F.D. Verbraak, MD PhD(PI), A.M.E. Schauwvlieghe, MD (reader), Douwe Bakker (reader)

Fundus Photograph Reading Center (Reading Centre, Moorfields Eye Hospital): Peto T, MD PhD(PI); Peter Blows (reader)

Committees

Data and Safety Monitoring Committee: Ringens PJ, MD, Phd (chair); R. Geskus (Biostastitician), MD;

R. Van Leeuwen,MD PhD

Funding

ZonMw The Netherlands Organisation for Health Research and Development

Edmond and Marianne Blaauw foundation for submission of the paper
