# Supplementary material for: Comparing the Effectiveness of Bevacizumab to Ranibizumab in Patients with Exudative Age-Related Macular Degeneration. The BRAMD Study
Source: PLoS One. 2016 May 20;11(5):e0153052. doi: 10.1371/journal.pone.0153052 (PMC4874598; doi:10.1371/journal.pone.0153052)
Supplement: S3 File — (DOC) [file pone.0153052.s003.doc]

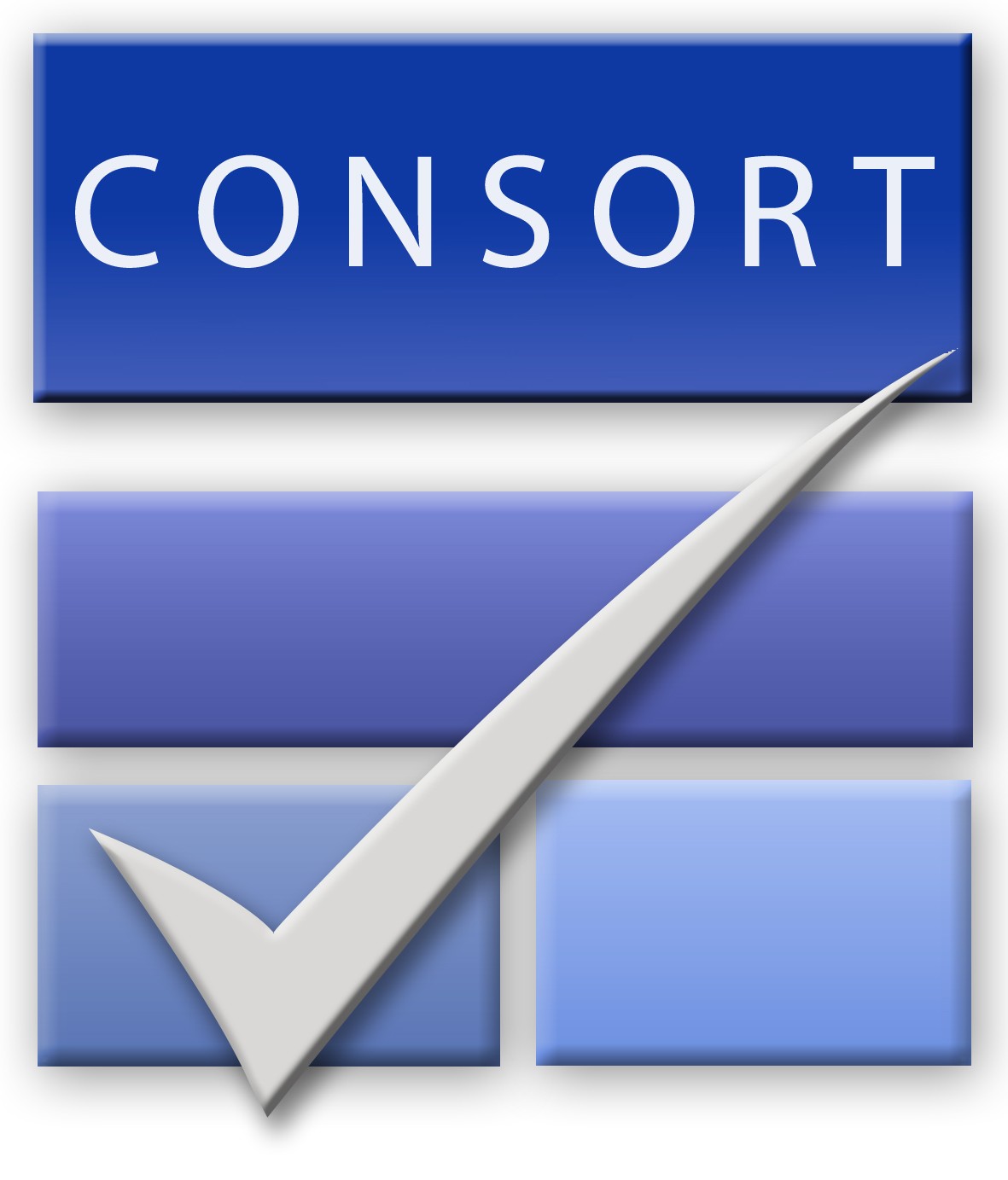
CONSORT 2010 checklist of information to include when reporting a randomised trial*

| Section/Topic | Item No | Checklist item | Reported on page No |
| --- | --- | --- | --- |
| Title and abstract | | | |
|  | 1a | Identification as a randomised trial in the title | The trial name is stated yet the title does not contain the words randomized clinical trial. The abstract does on page 2 |
|  | 1b | Structured summary of trial design, methods, results, and conclusions (for specific guidance see CONSORT for abstracts) | Yes, page 2 |
| Introduction | | | |
| Background and objectives | 2a | Scientific background and explanation of rationale | Yes, page 4-5 |
|  | 2b | Specific objectives or hypotheses | The introduction states it is a comparison between two drugs, on page 5 the methods section gives a more extensive explanation of the goals of the study on page 8 |
| Methods | | | |
| Trial design | 3a | Description of trial design (such as parallel, factorial) including allocation ratio | Yes page 5, line 123 |
|  | 3b | Important changes to methods after trial commencement (such as eligibility criteria), with reasons | No, though there were no significant changes in this section |
| Participants | 4a | Eligibility criteria for participants | Yes, page 5 line 123 to 131 and table 1 and 2 |
|  | 4b | Settings and locations where the data were collected | Page 5 and 7 |
| Interventions | 5 | The interventions for each group with sufficient details to allow replication, including how and when they were actually administered | Page 7 to 8, line 144-163 |
| Outcomes | 6a | Completely defined pre-specified primary and secondary outcome measures, including how and when they were assessed | Page 8-9, line 166 - 190 |
|  | 6b | Any changes to trial outcomes after the trial commenced, with reasons | No significant changes were made to the outcome measures |
| Sample size | 7a | How sample size was determined | Page 9 |
|  | 7b | When applicable, explanation of any interim analyses and stopping guidelines | A data safety monitoring board was set up. In case of safety issues the trial would be stopped, this is described in the protocol you can find in the supporting information though not in the manuscript itself |
| Randomisation: |  |  |  |
| Sequence generation | 8a | Method used to generate the random allocation sequence | Page 9, line 201-210 |
|  | 8b | Type of randomisation; details of any restriction (such as blocking and block size) | Page 9, line 201-210 |
| Allocation concealment mechanism | 9 | Mechanism used to implement the random allocation sequence (such as sequentially numbered containers), describing any steps taken to conceal the sequence until interventions were assigned | Page 7 and 9 |
| Implementation | 10 | Who generated the random allocation sequence, who enrolled participants, and who assigned participants to interventions | Page 9 for the allocation and assignment  Page 7, initial screening was done by the principal investigator at the participating sites |
| Blinding | 11a | If done, who was blinded after assignment to interventions (for example, participants, care providers, those assessing outcomes) and how | Page 9 |
|  | 11b | If relevant, description of the similarity of interventions | Page 7 |
| Statistical methods | 12a | Statistical methods used to compare groups for primary and secondary outcomes | Page 9 |
|  | 12b | Methods for additional analyses, such as subgroup analyses and adjusted analyses | Page 9 |
| Results | | | |
| Participant flow (a diagram is strongly recommended) | 13a | For each group, the numbers of participants who were randomly assigned, received intended treatment, and were analysed for the primary outcome | Page 11 and Figure 1 |
|  | 13b | For each group, losses and exclusions after randomisation, together with reasons | Page 11 and Figure 1 |
| Recruitment | 14a | Dates defining the periods of recruitment and follow-up | Page 11 |
|  | 14b | Why the trial ended or was stopped | Not stated, trial was completed |
| Baseline data | 15 | A table showing baseline demographic and clinical characteristics for each group | Table 2, page 11-12 |
| Numbers analysed | 16 | For each group, number of participants (denominator) included in each analysis and whether the analysis was by original assigned groups | Included in the tables 2 and 3 as well as the figures 2 and 3, and their legend |
| Outcomes and estimation | 17a | For each primary and secondary outcome, results for each group, and the estimated effect size and its precision (such as 95% confidence interval) | Table 3 page 15-16, page 13-17 |
|  | 17b | For binary outcomes, presentation of both absolute and relative effect sizes is recommended | Table 3 and in text |
| Ancillary analyses | 18 | Results of any other analyses performed, including subgroup analyses and adjusted analyses, distinguishing pre-specified from exploratory | Table 3 and page 15-17 |
| Harms | 19 | All important harms or unintended effects in each group (for specific guidance see CONSORT for harms) | Page 17-18 table 4 and 5 |
| Discussion | | | |
| Limitations | 20 | Trial limitations, addressing sources of potential bias, imprecision, and, if relevant, multiplicity of analyses | Page 21-22 |
| Generalisability | 21 | Generalisability (external validity, applicability) of the trial findings | Page 18-22 |
| Interpretation | 22 | Interpretation consistent with results, balancing benefits and harms, and considering other relevant evidence | Page 18-20 |
| Other information | | |  |
| Registration | 23 | Registration number and name of trial registry | Page 7 |
| Protocol | 24 | Where the full trial protocol can be accessed, if available | Supporting information file |
| Funding | 25 | Sources of funding and other support (such as supply of drugs), role of funders | Page 2 |

*We strongly recommend reading this statement in conjunction with the CONSORT 2010 Explanation and Elaboration for important clarifications on all the items. If relevant, we also recommend reading CONSORT extensions for cluster randomised trials, non-inferiority and equivalence trials, non-pharmacological treatments, herbal interventions, and pragmatic trials. Additional extensions are forthcoming: for those and for up to date references relevant to this checklist, see [www.consort-statement.org](http://www.consort-statement.org/).
